# Enhanced copper-resistance gene repertoire in *Alteromonas macleodii* strains isolated from copper-treated marine coatings

**DOI:** 10.1371/journal.pone.0257800

**Published:** 2021-09-28

**Authors:** Kathleen Cusick, Ane Iturbide, Pratima Gautam, Amelia Price, Shawn Polson, Madolyn MacDonald, Ivan Erill

**Affiliations:** 1 Department of Biological Sciences, University of Maryland Baltimore County, Baltimore, MD, United States of America; 2 Universitat Oberta de Catalunya, UOC, Barcelona, Spain; 3 Center for Bioinformatics and Computational Biology, University of Delaware, Newark, DE, United States of America; Westfalische Wilhelms-Universitat Munster, GERMANY

## Abstract

Copper is prevalent in coastal ecosystems due to its use as an algaecide and as an anti-fouling agent on ship hulls. *Alteromonas* spp. have previously been shown to be some of the early colonizers of copper-based anti-fouling paint but little is known about the mechanisms they use to overcome this initial copper challenge. The main models of copper resistance include the *Escherichia coli* chromosome-based Cue and Cus systems; the plasmid-based *E*. *coli* Pco system; and the plasmid-based *Pseudomonas syringae* Cop system. These were all elucidated from strains isolated from copper-rich environments of agricultural and/or enteric origin. In this work, copper resistance assays demonstrated the ability of *Alteromonas macleodii* strains CUKW and KCC02 to grow at levels lethal to other marine bacterial species. A custom database of Hidden Markov Models was designed based on proteins from the Cue, Cus, and Cop/Pco systems and used to identify potential copper resistance genes in CUKW and KCC02. Comparative genomic analyses with marine bacterial species and bacterial species isolated from copper-rich environments demonstrated that CUKW and KCC02 possess genetic elements of all systems, oftentimes with multiple copies, distributed throughout the chromosome and mega-plasmids. In particular, two copies of *copA* (the key player in cytoplasmic detoxification), each with its own apparent MerR-like transcriptional regulator, occur on a mega-plasmid, along with multiple copies of Pco homologs. Genes from both systems were induced upon exposure to elevated copper levels (100 μM– 3 mM). Genomic analysis identified one of the *merR-copA* clusters occurs on a genomic island (GI) within the plasmid, and comparative genomic analysis found that either of the *merR-copA* clusters, which also includes genes coding for a cupredoxin domain-containing protein and an isoprenylcysteine methyltransferase, occurs on a GI across diverse bacterial species. These genomic findings combined with the ability of CUKW and KCC02 to grow in copper-challenged conditions are couched within the context of the genome flexibility of the *Alteromonas* genus.

## Introduction

Copper is an essential trace metal required by living organisms ranging from bacteria to humans that serves as a cofactor in key enzymes involved in energy transduction, iron mobilization, and oxidative stress response [1, 2]. However, free copper is also toxic to living cells, as it interacts with protein thiol groups, competes with other metals for protein binding sites, and potentially generates reactive oxygen species [2]. Due to its biocidal effects, copper was used as an antimicrobial until the advent of commercially available antibiotics [3].

Bacteria have evolved multiple mechanisms by which to alleviate copper toxicity, including extracellular sequestration of copper ions, inner and outer membrane impermeability, metallothionein-like copper-scavenging proteins in both the cytoplasm and periplasm, and active export from the cell [3]. The primary models of copper resistance and homeostasis in Gram negative bacteria are derived from the *Escherichia coli* chromosome-based Cue and Cus systems; the plasmid-based *E*. *coli* Pco system; and the plasmid-based *Pseudomonas syringae* Cop system [4–6]. In general, the primary mechanism of cytoplasmic copper detoxification is via ATPase-driven copper efflux, while multicopper oxidases and RND transporters are the main components in periplasmic detoxification [5]. Most Gram-negative bacteria have at least one Cu-ATPase, commonly referred to as CopA, for cytoplasmic copper detoxification.

In *E*. *coli*, Cue (for copper efflux) is the primary resistance system for alleviating excess copper in the cytoplasm [6, 7]. CueO is a multicopper oxidase that may protect periplasmic enzymes from copper-induced damage [8], while CopA is a Cu(I)-translocating P-type ATPase [9]. The Cue system is regulated by the MerR-like transcriptional activator CueR, a one-component regulator that directly senses and responds to cytoplasmic copper [7, 10, 11]. Homologs of CueO include the plasmid-based multicopper oxidase PcoA of *E*. *coli*, along with the CopA multicopper oxidases of *Ralstonia metallidurans* and *P*. *syringae*. While none of these are P-type ATPases, they are necessary components of their respective copper systems and display similarity in sequence and function [12].

Cus (Cu-sensing) is a chromosomally-encoded, periplasmic efflux system consisting of RND transporters as well as a two-component signal transduction system [6]. The Cus system is comprised of two operons: (1.) the two-component signal transduction system *cusRS* that regulates *cusCFBA* and (2.) *cusCFBA*, whose members function in copper efflux [13, 14]. CusA is a member of the RND protein superfamily of proton-driven transporters and the main component of the multi-enzyme efflux pump [15, 16], while CusB is a membrane fusion protein [15], CusC an outer membrane factor (OMF) protein [17] and CusF a novel periplasmic chaperone [16]. This system, absent in Gram-positive bacteria, is exclusive to Gram-negative bacteria due to the need to transport metals across the outer membrane while protecting the periplasmic space from metal-induced damage [6].

Some microbes can survive and successfully colonize high-copper environments. The ability to overcome these copper challenges is typically achieved through plasmid, rather than chromosome, based systems. The two most well-studied plasmid-based systems come from Gram-negative bacteria typically associated with agricultural settings. The Pco (plasmid-borne copper resistance) system was found to reside on the 78KB conjugative plasmid Prj1004 in *E*. *coli* isolates from an Australian pig farm, in which CuSO_4_ was incorporated as dietary supplement. The Pco system is encoded by an operon containing six genes, *pcoABCDRS*, while a seventh gene, *pcoE*, is situated downstream of a copper-regulated promoter [18]. PcoA is a multicopper oxidase that, together with the periplasmic PcoC, are the primary components of the system, with PcoC binding and delivering cuprous ions to PcoA for subsequent oxidation. PcoB is an outer membrane protein, while PcoD is believed to be involved in copper uptake across the cytoplasmic membrane.

A plasmid-based copper resistance system, Cop, has also been identified from *Pseudomonas syringae* [19, 20]. The Cop system is comprised of six genes, *copABCDRS*, originally identified on plasmid Ppt23D [21]. CopA is a multicopper oxidase, while CopB is an outer membrane protein, and CopC a periplasmic chaperone with distinct regions for binding Cu(I) or Cu(II); and CopD is plasma membrane-embedded protein thought to transfer copper from CopC into the cytoplasm. The Cop and Pco systems are closely related based on both sequence similarity and Southern blot hybridization analyses [18, 22].

It is important to consider the environment or ecological niche inhabited by the various bacterial species for which copper resistance has been studied. *E*. *coli* is a facultative anaerobic enteric bacterium that inhabits the intestinal tract of mammalian and other warm-blooded animals. In this niche, copper likely does not exceed 10 μM, yet in the acidic conditions of some portions of the digestive tract, copper becomes more toxic; therefore, enteric bacteria may have evolved sophisticated copper homeostasis systems in response to their ecological niche (as discussed in [6]). The Pco system of *E*. *coli* was first identified in a strain isolated from the feces of pigs in Australia whose diet was supplemented with copper sulfate. Closely-related yet non-identical plasmids conferring copper resistance have been identified in several enteric species (*E*. *coli*, *Salmonella* sp., *Citrobacter* spp.) from a range of geographic locations [23]. Additionally, the Cop system was first identified in the plant pathogen *P*. *syringae*, where copper exposure occurs due to its application as an antifungal agent on tomato plants [19, 20].

Copper is prevalent in coastal ecosystems due to its use as an algaecide and as an anti-fouling (AF) agent on ship hulls [24, 25]. Copper-based (Cu_2_O) paints have become the main biocide of use in AF coatings due to their effectiveness, efficiency, and endurance following the ban of the tin-based, environmentally harmful compound tributyltin (TBT) by the International Maritime Organization [25, 26]. However, bacterial species that can overcome these copper challenges can then colonize the vessel surface, forming a biofilm that is the first step in the biofouling process. *Alteromonas* spp. have previously been shown to be some of the early colonizers of copper-based antifouling paint [27]; however, little is known about the mechanisms they use to overcome this initial copper challenge. An *A*. *macleodii* strain was previously isolated from Cu/Ni test coupons suspended in tropical coastal seawater [28]. Here, we report the closed genome sequence of this strain, CUKW, along with a strain (KCC02) created via continuous transfer in medium supplemented with 3 mM copper for one year. Putative copper resistance genes were identified and compared to those of other marine species as well as the model bacterial species for copper resistance systems. Expression profiling with a subset of copper genes demonstrated that CUKW and KCC02 utilize elements of multiple systems upon exposure to high copper levels, primarily two plasmid-based variants of *copA*. Phylogenetic and comparative genomic analyses found that either one or the other of the plasmid-based *copA* gene clusters in CUKW occurs in a genomic island across many bacterial species.

## Materials and methods

### Copper tolerance growth assays

The ability to grow at elevated copper levels was assessed in multiple marine bacterial species for comparison with *A*. *macleodii* strains CUKW and KCCO2 (Table 1). Copper (as CuSO_4_● H_2_O) (Sigma Aldrich, St. Louis, MO) was used as received. A 500 mM copper sulfate stock solution was prepared in deionized water, filter-sterilized through a 0.2 μM nitrocellulose filter, and stored in sterile, polycarbonate tubes. Single colonies of each strain were inoculated into 3 ml of Burkholder’s B Formulation or Difco marine broth 2216 (34.7 g per liter) and grown overnight with agitation (100 rpm) in sterile 50 ml tubes. Upon reaching late exponential phase, cultures were inoculated at a 1:100 dilution into 10 ml of fresh medium in sterile 50 ml tubes. Copper was added to final concentrations of 3 mM, 2 mM, 1 mM, or 100 μM. Controls consisted of sterile nuclease-free water added at the same volume as the copper solution. Abiotic controls consisted of cell-free medium to which was added the copper stock solution at final concentrations of 3 mM, 2 mM, 1 mM, 100 μm, and water only. Cultures were incubated with agitation (100 rpm) at 26° C. A minimum of two biological duplicates was performed at each copper concentration for each strain. Growth was defined as the change in optical density at 600 nm over time. The optical density was measured ca. every 4–6 h using the cuvette function of the Nanodrop 1C for 48–52 h.

**Table 1:** Marine species included in copper resistance assays.

Species	Growth Medium
*Alteromonas macleodii* CUKW	Burkholder’s B
*A*. *macleodii* KCC02	Burkholder’s B
*A*. *macleodii* 27126	Burkholder’s B
*Vibrio coralyticus* B183	Burkholder’s B
*V*. *alginolyticus*	Burkholder’s B
*V*. *harveyi*	Burkholder’s B
*Roseobacter denitrificans*	Burkholder’s B
*R*. *algicola*	Marine Broth 2216
*Pseudoalteromonas atlantica* TC6	Marine Broth 2216
*Ruegeria (Silicibacter)* TM1040	Burkholder’s B

### Genome sequencing: Culture conditions, DNA extraction and library preparation

*Alteromonas* strains CUKW and KCC02 were grown overnight in 5 ml Burkholder’s B medium with agitation (120 rpm). For each strain, two ml of the 5 ml culture was harvested for DNA extraction upon reaching mid-exponential phase (OD600 = ~1.4). DNA was extracted using the Masterpure Complete DNA and RNA purification kit (Lucigen) following a slightly modified version of the manufacturer’s protocol, in which vortexing was replaced by gentle re-suspension using wide-bore P1000 pipet tips in order to minimize DNA shearing.

### Sequencing, assembly, and annotation

DNA sequencing was performed using the PacBio platform. A gDNA Long Insert PacBio SMRTbell library was constructed for each strain and sequenced on a single-molecule real-time (SMRT) cell (P6-C4 chemistry, 240 min movie time) on a PacBio RS II instrument (Pacific Biosciences) at the University of Delaware. Assembly was performed using HGAP3 algorithm (CUKW settings: 1kb minimum subread/polymerase read length, 0.80 minimum polymerase read quality, 20kb minimum seed length; KCC02: 5kb minimum subread/polymerase read length, 0.85 minimum polymerase read quality, 20kb minimum seed length) in the PacBio SMRTpipe software (v. 2.3.0.139497) [29]. Chromosomes and plasmids were circularized using circulator (v. 1.5.3) [30].

The genomes were annotated by the University of Delaware Bioinformatics Core Facility using Prokka (v1.13.3) [31] with a custom annotation database including proteins from 28 *Alteromonas* species RefSeq genomes. Annotations and genome sequence were manually curated for consistency and to address occurrence of frameshifts in coding regions (*i*.*e*. pseudogenes).

### Identification and comparative genomics of copper-associated genes

Putative sequence homologs for the proteins of interest were identified using HMMER. A database of profile Hidden Markov Models (HMM) matching copper-associated genes from reference Cue, Cus, and Cop/Pco systems was compiled. These profile HMMs are summarized in Table 2. The proteomes of the species of interest were downloaded from NCBI in FASTA format and used as databases for the HMMER search, using the profile HMMs as a query (hmmsearch). The download and search process was automated with a Python script and the information obtained was stored in a comma separated value (csv) file. Raw data was then filtered to remove duplicate hits (hits that were obtained with more than one model as query), which were assigned to the query with the smallest e-value. This use of HMM model-based search methods was adopted as it allowed for a comprehensive comparison among bacterial genera rather than basing it on existing annotations or BLAST searches.

**Table 2:** Copper-associated protein models used in Hidden Markov Model database.

HMM Profile ID	Protein, Species, GenBank Accession No.
**TIGR02044**	CueR, *E*. *coli*, NP_415020.1
**TIGR01480**	CueO, *E*. *coli*, NP_414665.1
	PcoA, *E*. *coli*, ANH09828.1
	CopA, *Pseudomonas syringae* pv. *Syringae*, AQX42270.1
**COG2217**	CopA, *E*. *coli*, NP_415017.1
**TIGR01386**	CusS, *E*. *coli*, NP_415102.1
	PcoS, *E*. *coli*, ANH09782.1
	CopS, *P*. *syringae*, AQX42266.1
**TIGR01387**	CusR, *E*. *coli*, NP_415103.1
	PcoR, *E*. *coli*, ANH09781.1
	CopR, *P*. *syringae*, AQX42267.1
**COG3696**	CusA, *E*. *coli*, NP_415107.1
**COG0845**	CusB, *E*. *coli*, NP_415106.1
**TIGR01845**	CusC, *E*. *coli*, NP_415104.1
**COG5569**	CusF, *E*. *coli*, NP_415105.1
**COG3667**	PcoB, *E*. *coli*, ANH09778.1
	CopB, *P*. *syringae*, AQX42189.1
**COG2372**	PcoC, *E*. *coli*, ANH09779.1
	CopC, *P*. *syringae*, AQX42188.1
**COG1276**	PcoD, *E*. *coli*, ANH09780.1
	CopD, *P*. *syringae*, AQX42268.1
**PF11106.8**	PcoE, *E*. *coli*, ANH09783.1
	PcoF, *E*. *coli*, AFX60851.1
**COG0739**	PcoG, *E*. *coli*, AZZ87773.1
**TIGR00003**	CopZ, *P*. *syringae*, AQX41994.1
**TIGR02698**	CopY, *Streptococcus mutans*, AAG10085.1
**COG1937**	CsoR, *Staphylococcus haemolyticus*, AMP34391.1

For visualization of the data and exploratory analysis RStudio was used. The number of hits per protein and per species were counted, grouping each time for the category of interest. Data was visualized using the ggplot2 library.

The neighborhoods surrounding the putative copper resistance genes of CUWK were further analyzed, including adjacent regulatory elements, potential co-localization of resistance genes, and presence of mobile genetic elements. GC content analysis and visualization of genome arrangement of chromosome and plasmids was performed in Artemis. Island Viewer (http://www.pathogenomics.sfu.ca/islandviewer/, [32]) was used to identify genomic islands in the CUKW genome and pCUKW-178. Genomic neighborhoods for specific copper resistance genes were further investigated using the Enzyme Function Initiative Genome Neighborhood Network server using default settings for the Enzyme Similarity Tool and the Genome Neighborhood Tool [33, 34].

### Expression profiling with a subset of copper-associated genes

A subset of genes identified from genome annotation using the Prokka pipeline were selected for expression profiling based on their homology to the copper-translocating ATPase *copA* gene or the *pco/cop* genes. Two *copA* variants were found to occur on pCUKW-178 and pKCC02-243 (discussed in detail below), each with a putative *merR* regulator immediately upstream, and so these two regulators were also included in the transcriptional analysis. Single colonies were grown O/N in 3 ml Burkholder’s B medium to an OD = 1.8. 1:100 dilutions were inoculated into 10 ml Burkholder’s B medium in 30 ml glass culture tubes and incubated under static conditions at 28° C for 6 h, whereupon copper (as CuS0_4_.H_2_0) was added to final concentrations of 100 μM, 1 mM, and 3 mM. Controls consisted of nuclease-free water added at the same volume as the copper solution. Cultures were incubated statically for 30 min, vortexed briefly, and 2 ml removed for RNA extraction. Cultures were then incubated another 90 min, vortexed briefly, and 2 ml removed for RNA extraction. Cells were pelleted by centrifugation at room temp for 2 min at 10,000 g, washed once in PBS, centrifuged, and re-suspended in 40 μl RNALater. Tubes were stored at -80° C until RNA extraction.

### Total RNA extraction and cDNA synthesis

Prior to RNA extraction, the pellets were thawed and the RNALater removed. Total RNA was extracted as described previously [28] using a modified protocol of the RNeasy mini kit. Total RNA concentration and purity were assessed using the Nanodrop 1C. Total RNA was converted to cDNA using the High-Capacity RNA-to-cDNA kit (Applied Biosystems, Foster City, CA). Each reaction contained 10 μl 2x RT buffer, 1 μl 20x enzyme, 2 μg total RNA, and brought to a final volume of 20 μl with nuclease-free water. No-RT controls consisted of all components except the enzyme. Reactions were performed in the ProFlex thermocycler (Applied Biosystems) under the following conditions: 37° C for 60 min, followed by 95° C for 5 min. Samples were diluted 1:10 in nuclease-free water prior to use in quantitative PCR.

RT-qPCR assays based on SYBR Green chemistry were designed to examine the multiple copies of the *copA* as well as homologs within the *P*. *syringae* Cop system (Table 3). For each assay, the genome sequences of CUKW and KCC02 were aligned, and primers designed that targeted a conserved region of the gene spanning 100–130 bp in length. Primers were designed using the Primer 3 software [35, 36] with an optimal annealing temperature of 60° C. The potential for secondary structures was examined using Mfold [37], while dimer formation and self-complementarity were examined using the oligonucleotides properties calculator (http://www.basic.northwestern.edu/biotools/oligocalc.html). Assays were optimized over primer concentrations spanning 150–300 nM. Product specificity was initially assessed using gel electrophoresis and confirmed with melt curve analysis. The reference genes *maf*, *inf2*, and *pfk*, previously designed and optimized [28], were assessed for stability under the different copper conditions using the program BestKeeper [38]. All displayed nearly equal stability values (as assessed by SD, standard deviation of the C_T;_ in which values <1 are considered stable [*maf* = 0.6, *inf2* = 0.44, *pfk* = 0.52), and so *pfk* was selected for use based on the recommendation of reference genes being expressed at comparable levels to target genes [39]. The PowerUp SYBR Green Master Mix (Applied Biosystems, Foster City, CA) was used for all assays. Each reaction contained: 10 μL 2x PowerUp SYBR Green master mix, 150–300 nM each forward and reverse primer, 2 μL of template cDNA diluted 1:10, and brought to a final volume of 20 μL with nuclease-free water. Reactions were performed on the QuantStudio 6 Real Time PCR System with the 96-well block format (Applied Biosystems, Foster City, CA). The following protocol was used for all assays: an initial 20 s incubation at 95° C, followed by 40 cycles of 95° C for 1 s and 60° C for 20 s, followed by a melt curve analysis of 95° C for 15 s, 60° C for 1 min, and 95° C for 15 s to determine product specificity. All qPCR reactions were performed in duplicate using Applied Biosystems MicroAmp Fast 96-well reaction plates sealed with MicroAmp optical adhesive film. No-template controls were also included in each amplification run to monitor for contamination. Reactions were recorded and analyzed using the Applied Biosystems QuantStudio 6 System software. Gene expression following 30 min and 2 h copper exposure was calculated using the ΔΔC_T_ method [40], with cultures to which nuclease-free water (the carrier of the copper solution) was added at the same volume as the copper solution serving as the controls.

**Table 3:** Copper-associated genes used in expression profiling.

CUKW Locus ID	KCC02 Locus ID	Description	Primer Sequence
CUKW_02050	KCCO2_02059	Cu-translocating P-type ATPase *(E*. *coli*)	F: GCAAGTCGCGGTGAATGTAC
			R: GTGTTCGTCGGGGTGAAATG
CUKW_04347	KCC02_04215	MerR transcriptional regulator	F: CACGTCATCACAAACCAACG
			R: GCGCAGTCTTAGCTCATGC
CUKW_04348	KCCO2_04216	Cu-translocating P-type ATPase (*E*. *coli*)	F: GTGCTTTCTTTGGCGGCTAG
			R: GGAATGGATCGGCACACCTT
CUKW_04383	KCC02_04252	MerR transcriptional regulator	F: CCGCTTGTCCCCTAGTTAGA
			R: CATTGCTCCCCATTTCCTTA
CUKW_04384	KCCO2_04253	Cu-translocating P-type ATPase (*E*. *coli*)	F: ACGAGTCAATGCTAACCGGG
			R: CCAGTGCCGTGTCTTTACCT
CUKW_04393	KCC02_04262	*P*. *syringae* copper resistance A; multicopper oxidase	F: CCCCACAGCGAGTTATCACA
			R: TGCTGCCCCATTCCTTTCAT
CUKW_04392	KCC02_04261	*P*. *syringae* copper resistance B	F: GCGTGGTATGGCGGAGATTA
			R: ACAACAGTTCTGCTCGTTCCA
CUKW_04378	KCC02_04247	*P*. *syringae* copper resistance D	F: AGGTTTGTGTCTTTGTTGCGT
			R: CATAAAGGGGTCAAGTGCGC
CUKW_04377	KCC02_04246	*P*. *syringae* copper resistance C	F: CGAAAGAGGTTGCAACGGATT
			R: CCATCGGAGCCAAGCATGA
CUKW_04318	KCC02_04186	*P*. *syringae* copper resistance D	F: TGCTTGGTGGTGGTTGGATT
			R: AATGACCGCAAACAAACGCA
CUKW_04317	KCC02_04185	*P*. *syringae* copper resistance C	F: GTTGCTAGGTCAGGACGGTC
			R: CTAATGGTTGCCGTGCGTG

### Statistical analysis of gene expression data

The ΔC_T_ was calculated for each biological replicate for each gene under each condition and examined for normal distribution using the Shapiro-Wilkes test. If data were normally distributed, t-tests were used to test for significant differences between copper and no-copper treatments at each concentration and time point for each strain. If the data were not normally distributed, the non-parametric Mann-Whitney U-test was applied.

### *copA* characterization and distribution

The annotation derived from the Prokka and RefSeq pipelines was used for genomic analysis, including sequence diversity, genome architecture, and gene content in CUKW and comparative genomic analyses among *Alteromonas* sp. for *copA* and *merR* variants. As KCC02 had nearly identical *copA* sequences and genome architecture, only CUKW is described here. A BLASTN search was performed with the sequence of the two *copA* variants on pCUKW-178 and all publicly available closed genomes of *A*. *macleodii* and *A*. *mediterranea* strains to identify the presence and sequence identity of either variant. A local BLASTP analysis was also performed on these genomes as well as closed genomes for other *Alteromonas* species (Table 4). To identify CopA homologs in other bacterial species, we searched for homologous protein sequences in GenBank using BLASTP [41], using the amino acid sequences of the two CopA variants as the queries and retaining sequences that were greater than 45% similar at the amino acid level and between 40% and 150% the length of the query, per the parameters previously used in the identification of bacterial copper resistance genes [42]. To identify the CopA homolog and the gene cluster surrounding it, a local BLASTP analysis was performed using Biopython. The amino acid sequence of CopA variants along with the MerR transcriptional regulator, a cupredoxin domain-containing protein, an isoprenylcysteine methyltransferase and a hypothetical protein from CUKW were used as queries for BLASTP analysis using the same parameters as described above for CopA. The identical protein group (ipg) records for each blast hit were accessed to select the protein sequences from complete genome records only. A phylogenetic analysis was performed based on the amino acid sequences for all CopA variants retrieved from the BLASTP analysis. The protein record, locus id, functional description for the *copA* variants and the gene cluster for each species is listed in Table 5. Island Viewer (http://www.pathogenomics.sfu.ca/islandviewer/, [32]) was used to identify genomic islands within the genomes of *Alteromonas* spp. and all other species included in the *copA* gene cluster analysis for comparison with CUKW.

**Table 4:** Presence and genome organization of *merR-copA* clusters in *Alteromonas*.

Species	Locus ID	Protein ID	Description	On GI
*Alteromonas* sp. MB-3u-76 (NZ_CP025115.1)	CW735_RS08630	WP_100971716.1	MerR family transcriptional regulator	Y
*Alteromonas* sp. MB-3u-76 (NZ_CP025115.1)	CW735_RS08635	WP_100973221.1	copper-translocating P-type ATPase	Y
*Alteromonas* sp. MB-3u-76 (NZ_CP025115.1)	CW735_RS08640	WP_044447459.1	cupredoxin domain-containing protein	Y
*Alteromonas* sp. MB-3u-76 (NZ_CP025115.1)	CW735_RS08645	WP_013755268.1	DUF2933 domain-containing protein	Y
*Alteromonas* sp. MB-3u-76 (NZ_CP025115.1)	CW735_RS08650	WP_013755267.1	isoprenylcysteine carboxylmethyltransferase	Y
*Alteromonas* sp. RW2A1 (NZ_CP018031.1)	BM528_RS05075	WP_044447457.1	MerR family DNA-binding protein	Y
*Alteromonas* sp. RW2A1 (NZ_CP018031.1)	BM528_RS05080	WP_071981165.1	copper-translocating P-type ATPase	Y
*Alteromonas* sp. RW2A1 (NZ_CP018031.1)	BM528_RS05085	WP_044447459.1	cupredoxin domain-containing protein	Y
*Alteromonas* sp. RW2A1 (NZ_CP018031.1)	BM528_RS05090	WP_013755268.1	DUF2933 domain-containing protein	Y
*Alteromonas* sp. RW2A1 (NZ_CP018031.1)	BM528_RS05095	WP_071978954.1	isoprenylcysteine carboxylmethyltransferase	Y
*Alteromonas* sp. RKMC-009 (NZ_CP031010.1)	DS731_RS05430	WP_119500363.1	MerR family transcriptional regulator	N
*Alteromonas* sp. RKMC-009 (NZ_CP031010.1)	DS731_RS05435	WP_119500364.1	copper-translocating P-type ATPase	N
*Alteromonas* sp. RKMC-009 (NZ_CP031010.1)	DS731_RS05440	WP_119500365.1	cupredoxin domain-containing protein	N
*Alteromonas* sp. RKMC-009 (NZ_CP031010.1)	DS731_RS05445	WP_119500366.1	DUF2933 domain-containing protein	N
*Alteromonas* sp. RKMC-009 (NZ_CP031010.1)	DS731_RS05450	WP_119500367.1	isoprenylcysteine carboxylmethyltransferase	N
*A*. *macleodii* HOT1A3 (NZ_CP012203.1)	ACZ81_RS20215	WP_012516866.1	MerR family DNA-binding protein	N*
*A*. *macleodii* HOT1A3 (NZ_CP012203.1)	ACZ81_RS20210	None	copper-translocating P-type ATPase	N*
*A*. *macleodii* HOT1A3 (NZ_CP012203.1)	ACZ81_RS20205	WP_012516868.1	cupredoxin domain-containing protein	N*
*A*. *macleodii* HOT1A3 (NZ_CP012203.1)	ACZ81_RS20200	WP_012516869.1	DUF2933 domain-containing protein	N*
*A*. *macleodii* HOT1A3 (NZ_CP012203.1)	ACZ81_RS20195	None	isoprenylcysteine carboxylmethyltransferase	N*
*A*. *macleodii* HOT1A3 (NZ_CP012203.1)	ACZ81_RS20705	WP_012516906.1	MerR family DNA-binding protein	N*
*A*. *macleodii* HOT1A3 (NZ_CP012203.1)	ACZ81_RS20710	WP_012516907.1	copper-translocating P-type ATPase	N*
*A*. *macleodii* HOT1A3 (NZ_CP012203.1)	ACZ81_RS20715	WP_012516908.1	cupredoxin domain-containing protein	N*
*A*. *macleodii* HOT1A3 (NZ_CP012203.1)	ACZ81_RS20720	WP_012516909.1	DUF2933 domain-containing protein	N*
*A*. *macleodii* HOT1A3 (NZ_CP012203.1)	ACZ81_RS20725	None	isoprenylcysteine carboxylmethyltransferase	N*
*Alteromonas* sp. 76–1 (NZ_LR136958.1)	ALT761_RS10760	WP_032094223.1	isoprenylcysteine carboxylmethyltransferase	N
*Alteromonas* sp. 76–1 (NZ_LR136958.1)	ALT761_RS10765	WP_032094224.1	DUF2933 domain-containing protein	N
*Alteromonas* sp. 76–1 (NZ_LR136958.1)	ALT761_RS10770	WP_172602873.1	cupredoxin domain-containing protein	N
*Alteromonas* sp. 76–1 (NZ_LR136958.1)	ALT761_RS10775	WP_172602874.1	copper-translocating P-type ATPase	N
A. *australica* DE170 (NZ_CP010912.1)	EP12_RS01540	WP_012516906.1	MerR family transcriptional regulator	Y
*A*. *australica* DE170 (NZ_CP010912.1)	EP12_RS01545	WP_012516907.1	cation transport ATPase	Y
*A*. *australica* DE170 (NZ_CP010912.1)	EP12_RS01550	WP_012516908.1	protein containing plastocyanin domain	Y
*A*. *australica* DE170 (NZ_CP010912.1)	EP12_RS01555	WP_012516909.1	hypothetical protein	Y
*A*. *australica* DE170 (NZ_CP010912.1)	EP12_RS01560	WP_024015555.1	protein-S-isoprenylcysteine methyltransferase	Y
*A*. *australica* DE170 (NZ_CP010912.1)	EP12_RS10150	WP_024015555.2	MerR family transcriptional regulator	N
*A*. *australica* DE170 (NZ_CP010912.1)	EP12_RS10155	WP_024015555.3	copper-translocating P-type ATPase	N
*A*. *australica* DE170 (NZ_CP010912.1)	EP12_RS10160	WP_024015555.4	plastocyanin	N
*A*. *australica* DE170 (NZ_CP010912.1)	EP12_RS10165	WP_024015555.5	hypothetical protein	N
*A*. *australica* DE170 (NZ_CP010912.1)	EP12_RS10170	WP_024015555.6	isoprenylcysteine carboxyl methyltransferase	N
*A*. *naphthalenivorans* (NC_015554.1)	AMBT_RS07080	WP_024015555.7	copper-translocating P-type ATPase	Y
*A*. *naphthalenivorans* (NC_015554.1)	AMBT_RS07085	WP_024015555.8	plastocyanin	Y
*A*. *naphthalenivorans* (NC_015554.1)	AMBT_RS07090	WP_024015555.9	hypothetical protein	Y
*A*. *naphthalenivorans* (NC_015554.1)	AMBT_RS07095	WP_024015555.10	isoprenylcysteine carboxyl methyltransferase	Y
*A*. *macleodii* Te101 (NZ_CP018321.1)	TE101_RS01270	WP_024015555.11	MerR family DNA-binding transcriptional regulator	Y
*A*. *macleodii* Te101 (NZ_CP018321.1)	TE101_RS01275	WP_024015555.12	copper-translocating P-type ATPase	Y
*A*. *macleodii* Te101 (NZ_CP018321.1)	TE101_RS01280	WP_024015555.13	cupredoxin domain-containing protein	Y
*A*. *macleodii* Te101 (NZ_CP018321.1)	TE101_RS01285	WP_024015555.14	DUF2933 domain-containing protein	Y
*A*. *macleodii* Te101 (NZ_CP018321.1)	TE101_RS01290	WP_024015555.15	isoprenylcysteine carboxylmethyltransferase	Y
*A*. *macleodii* D7 (NZ_CP014323.1)	AVL55_RS01240	WP_024015555.16	Zn(II)-responsive transcriptional regulator	Y
*A*. *macleodii* D7 (NZ_CP014323.1)	AVL55_RS01245	WP_024015555.17	Cu+ exporting ATPase	Y
*A*. *macleodii* D7 (NZ_CP014323.1)	AVL55_RS01250	WP_024015555.18	cupredoxin domain-containing protein	Y
*A*. *macleodii* D7 (NZ_CP014323.1)	AVL55_RS01255	WP_024015555.19	hypothetical protein	Y
*A*. *macleodii* D7 (NZ_CP014323.1)	AVL55_RS01260	WP_024015555.20	isoprenylcysteine carboxyl methyltransferase	Y
*A*. *macleodii* D7 (NZ_CP014323.1)	AVL55_RS01385	WP_024015555.21	MerR family transcriptional regulator	Y
*A*. *macleodii* D7 (NZ_CP014323.1)	AVL55_RS01390	WP_024015555.22	cation transport ATPase	Y
*A*. *macleodii* D7 (NZ_CP014323.1)	AVL55_RS01395	WP_024015555.23	protein containing plastocyanin domain	Y
*A*. *macleodii* D7 (NZ_CP014323.1)	AVL55_RS01400	WP_024015555.24	hypothetical protein	Y
*A*. *macleodii* D7 (NZ_CP014323.1)	AVL55_RS01405	WP_024015555.25	isoprenylcysteine carboxyl methyltransferase	Y
*A*. *macleodii* D7 (NZ_CP014323.1)	AVL55_RS05865	WP_024015555.26	ATPase	Y
*A*. *macleodii* Black Sea 11 (NC_018692.1)	AMBLS11_RS01365	WP_024015555.27	MerR family transcriptional regulator	Y
*A*. *macleodii* Black Sea 11 (NC_018692.1)	AMBLS11_RS01370	WP_024015555.28	copper-transporting ATPase	Y
*A*. *macleodii* Black Sea 11 (NC_018692.1)	AMBLS11_RS01375	WP_024015555.29	cupredoxin domain-containing protein	Y
*A*. *macleodii* Black Sea 11 (NC_018692.1)	AMBLS11_RS01380	WP_024015555.30	hypothetical protein	Y
*A*. *macleodii* Black Sea 11 (NC_018692.1)	AMBLS11_RS01385	WP_024015555.31	isoprenylcysteine carboxyl methyltransferase	Y
*A*. *mediterranea* UM8 (NZ_CP013928.1)	AV942_RS01540	WP_024015555.32	Zn(II)-responsive transcriptional regulator	Y
*A*. *mediterranea* UM8 (NZ_CP013928.1)	AV942_RS01545	WP_024015555.33	copper-transporting ATPase	Y
*A*. *mediterranea* UM8 (NZ_CP013928.1)	AV942_RS01550	WP_024015555.34	plastocyanin	Y
*A*. *mediterranea* UM8 (NZ_CP013928.1)	AV942_RS01555	WP_024015555.35	hypothetical protein	Y
*A*. *mediterranea* UM8 (NZ_CP013928.1)	AV942_RS01560	WP_024015555.36	isoprenylcysteine carboxyl methyltransferase	Y
*A*. *mediterranea* UM8 (NZ_CP013928.1)	AV942_RS01695	WP_024015555.37	MerR family transcriptional regulator	Y
*A*. *mediterranea* UM8 (NZ_CP013928.1)	AV942_RS01700	WP_024015555.38	cation transport ATPase	Y
*A*. *mediterranea* UM8 (NZ_CP013928.1)	AV942_RS01705	WP_024015555.39	protein containing plastocyanin domain	Y
*A*. *mediterranea* UM8 (NZ_CP013928.1)	AV942_RS01710	WP_024015555.40	hypothetical protein	Y
*A*. *mediterranea* UM8 (NZ_CP013928.1)	AV942_RS01715	WP_024015555.41	protein-S-isoprenylcysteine methyltransferase	Y
*A*. *mediterranea* UM8 (NZ_CP013928.1)	AV942_RS05830	WP_024015555.42	ATPase	Y
*A*. *mediterranea* DE (NC_011138.3)	MADE_RS01685	WP_024015555.43	MerR family transcriptional regulator	Y
*A*. *mediterranea* DE (NC_011138.3)	MADE_RS01690	WP_024015555.44	copper-transporting ATPase	Y
*A*. *mediterranea* DE (NC_011138.3)	MADE_RS01695	WP_024015555.45	plastocyanin	Y
*A*. *mediterranea* DE (NC_011138.3)	MADE_RS01700	WP_024015555.46	hypothetical protein	Y
*A*. *mediterranea* DE (NC_011138.3)	MADE_RS01705	WP_024015555.47	isoprenylcysteine carboxyl methyltransferase	Y
*A*. *mediterranea* DE (NC_011138.3)	MADE_RS01875	WP_024015555.48	MerR family transcriptional regulator	Y
*A*. *mediterranea* DE (NC_011138.3)	MADE_RS01880	WP_024015555.49	cation transport ATPase	Y
*A*. *mediterranea* DE (NC_011138.3)	MADE_RS01885	WP_024015555.50	protein containing plastocyanin domain	Y
*A*. *mediterranea* DE (NC_011138.3)	MADE_RS01890	WP_024015555.51	hypothetical protein	Y
*A*. *mediterranea* DE (NC_011138.3)	MADE_RS01895	WP_024015555.52	isoprenylcysteine carboxyl methyltransferase	Y
*A*. *mediterranea* DE1 (NC_019393.1)	AMAD1_RS01550	WP_024015555.53	Zn(II)-responsive transcriptional regulator	Y
*A*. *mediterranea* DE1 (NC_019393.1)	AMAD1_RS01555	WP_024015555.54	copper-transporting ATPase	Y
*A*. *mediterranea* DE1 (NC_019393.1)	AMAD1_RS01560	WP_024015555.55	plastocyanin	Y
*A*. *mediterranea* DE1 (NC_019393.1)	AMAD1_RS01565	WP_024015555.56	hypothetical protein	Y
*A*. *mediterranea* DE1 (NC_019393.1)	AMAD1_RS01570	WP_024015555.57	isoprenylcysteine carboxyl methyltransferase	Y
*A*. *mediterranea* DE1 (NC_019393.1)	AMAD1_RS01710	WP_024015555.58	MerR family transcriptional regulator	Y
*A*. *mediterranea* DE1 (NC_019393.1)	AMAD1_RS01715	WP_024015555.59	cation transport ATPase	Y
*A*. *mediterranea* DE1 (NC_019393.1)	AMAD1_RS01720	WP_024015555.60	protein containing plastocyanin domain	Y
*A*. *mediterranea* DE1 (NC_019393.1)	AMAD1_RS01725	WP_024015555.61	hypothetical protein	Y
*A*. *mediterranea* DE1 (NC_019393.1)	AMAD1_RS01730	WP_024015555.62	protein-S-isoprenylcysteine methyltransferase	Y
*A*. *mediterranea* DE1 (NC_019393.1)	AMAD1_RS05875	WP_024015555.63	ATPase	Y
*A*. *mediterranea* UM7 (NC_021713.1)	I635_RS01545	WP_024015555.64	Zn(II)-responsive transcriptional regulator	Y
*A*. *mediterranea* UM7 (NC_021713.1)	I635_RS01550	WP_024015555.65	copper-transporting ATPase	Y
*A*. *mediterranea* UM7 (NC_021713.1)	I635_RS01555	WP_024015555.66	plastocyanin	Y
*A*. *mediterranea* UM7 (NC_021713.1)	I635_RS01560	WP_024015555.67	hypothetical protein	Y
*A*. *mediterranea* UM7 (NC_021713.1)	I635_RS01565	WP_024015555.68	isoprenylcysteine carboxyl methyltransferase	Y
*A*. *mediterranea* UM7 (NC_021713.1)	I635_RS01705	WP_024015555.69	MerR family transcriptional regulator	Y
*A*. *mediterranea* UM7 (NC_021713.1)	I635_RS01710	WP_024015555.70	cation transport ATPase	Y
*A*. *mediterranea* UM7 (NC_021713.1)	I635_RS01715	WP_024015555.71	protein containing plastocyanin domain	Y
*A*. *mediterranea* UM7 (NC_021713.1)	I635_RS01720	WP_024015555.72	hypothetical protein	Y
*A*. *mediterranea* UM7 (NC_021713.1)	I635_RS01725	WP_024015555.73	protein-S-isoprenylcysteine methyltransferase	Y
*A*. *mediterranea* UM4b (NC_021714.1)	I636_RS01535	WP_024015555.75	Zn(II)-responsive transcriptional regulator	Y
*A*. *mediterranea* UM4b (NC_021714.1)	I636_RS01540	WP_024015555.76	copper-transporting ATPase	Y
*A*. *mediterranea* UM4b (NC_021714.1)	I636_RS01545	WP_024015555.77	plastocyanin	Y
*A*. *mediterranea* UM4b (NC_021714.1)	I636_RS01550	WP_024015555.78	hypothetical protein	Y
*A*. *mediterranea* UM4b (NC_021714.1)	I636_RS01555	WP_024015555.79	isoprenylcysteine carboxyl methyltransferase	Y
*A*. *mediterranea* UM4b (NC_021714.1)	I636_RS01685	WP_024015555.80	MerR family transcriptional regulator	Y
*A*. *mediterranea* UM4b (NC_021714.1)	I636_RS01690	WP_024015555.81	cation transport ATPase	Y
*A*. *mediterranea* UM4b (NC_021714.1)	I636_RS01695	WP_024015555.82	protein containing plastocyanin domain	Y
*A*. *mediterranea* UM4b (NC_021714.1)	I636_RS01700	WP_024015555.83	hypothetical protein	Y
*A*. *mediterranea* UM4b (NC_021714.1)	I636_RS01705	WP_024015555.84	protein-S-isoprenylcysteine methyltransferase	Y
*A*. *mediterranea* UM4b (NC_021714.1)	I636_RS05880	WP_024015555.85	ATPase	Y
*A*. *mediterranea* MED64 (NC_023045.1)	I533_RS01485	WP_024015555.86	Zn(II)-responsive transcriptional regulator	Y
*A*. *mediterranea* MED64 (NC_023045.1)	I533_RS01490	WP_024015555.87	copper-transporting ATPase	Y
*A*. *mediterranea* MED64 (NC_023045.1)	I533_RS01495	WP_024015555.88	plastocyanin	Y
*A*. *mediterranea* MED64 (NC_023045.1)	I533_RS01500	WP_024015555.89	hypothetical protein	Y
*A*. *mediterranea* MED64 (NC_023045.1)	I533_RS01505	WP_024015555.90	isoprenylcysteine carboxyl methyltransferase	Y
*A*. *mediterranea* MED64 (NC_023045.1)	I533_RS01665	WP_024015555.91	MerR family transcriptional regulator	Y
*A*. *mediterranea* MED64 (NC_023045.1)	I533_RS01670	WP_024015555.92	cation transport ATPase	Y
*A*. *mediterranea* MED64 (NC_023045.1)	I533_RS01675	WP_024015555.93	protein containing plastocyanin domain	Y
*A*. *mediterranea* MED64 (NC_023045.1)	I533_RS01680	WP_024015555.94	hypothetical protein	Y
*A*. *mediterranea* MED64 (NC_023045.1)	I533_RS01685	WP_024015555.95	protein-S-isoprenylcysteine methyltransferase	Y
*A*. *mediterranea* U7 (NC_021717.1)	I876_RS01780	WP_024015555.96	Zn(II)-responsive transcriptional regulator	Y
*A*. *mediterranea* U7 (NC_021717.1)	I876_RS01785	WP_024015555.97	copper-transporting ATPase	Y
*A*. *mediterranea* U7 (NC_021717.1)	I876_RS01790	WP_024015555.98	plastocyanin	Y
*A*. *mediterranea* U7 (NC_021717.1)	I876_RS01795	WP_024015555.99	hypothetical protein	Y
*A*. *mediterranea* U7 (NC_021717.1)	I876_RS01800	WP_024015555.100	isoprenylcysteine carboxyl methyltransferase	Y
*A*. *macleodii* Balearic Sea AD45 (NC_018679.1)	AMBAS45_RS01335	WP_024015555.101	MerR family transcriptional regulator	Y
*A*. *macleodii* Balearic Sea AD45 (NC_018679.1)	AMBAS45_RS01340	WP_024015555.102	cation transport ATPase	Y
*A*. *macleodii* Balearic Sea AD45 (NC_018679.1)	AMBAS45_RS01345	WP_024015555.103	protein containing plastocyanin domain	Y
*A*. *macleodii* Balearic Sea AD45 (NC_018679.1)	AMBAS45_RS01350	WP_024015555.104	hypothetical protein	Y
*A*. *macleodii* Balearic Sea AD45 (NC_018679.1)	AMBAS45_RS01355	WP_024015555.105	isoprenylcysteine carboxyl methyltransferase	Y

* after Y or N denotes it occurs on a plasmid.

**Table 5:** *copA* cluster presence and genome organization across genera.

Species	Locus ID	Protein ID	Description	On GI
*Vibrio furnissii* NCTC 11218 (NC_016602.1)	VFU_RS01050	WP_014204102.1	cation transport ATPase	Y
*V*. *furnissii* NCTC 11218	VFU_RS01055	WP_041942450.1	plastocyanin	Y
*V*. *furnissii* NCTC 11218	VFU_RS01060	WP_014204103.1	hypothetical protein	Y
*V*. *furnissii* NCTC 11218	VFU_RS01065	WP_014204104.1	isoprenylcysteine carboxylmethyltransferase	Y
*V*. *furnissii* 2013V-1001 (NZ_CP046797.1)	GPY43_RS14250	WP_014204104.1	isoprenylcysteine carboxylmethyltransferase	Y
*V*. *furnissii* 2013V-1001 (NZ_CP046797.1)	GPY43_RS14255	WP_014204103.1	DUF2933 domain-containing protein	Y
*V*. *furnissii* 2013V-1001 (NZ_CP046797.1)	GPY43_RS14260	WP_041942450.1	cupredoxin domain-containing protein	Y
*V*. *furnissii* 2013V-1001 (NZ_CP046797.1)	GPY43_RS14265	WP_014204102.1	copper-translocating P-type ATPase	Y
*Pseudoalteromonas spongiae* (NZ_CP023398.1)	SAO4_RS03140	WP_085281994.1	MerR family DNA-binding transcriptional regulator	Y
*P*. *spongiae* (NZ_CP023398.1)	SAO4_RS03145	WP_100912775.1	copper-translocating P-type ATPase	Y
*P*. *spongiae* (NZ_CP023398.1)	SAO4_RS03150	WP_100912776.1	plastocyanin	Y
*P*. *spongiae* (NZ_CP023398.1)	SAO4_RS03160	WP_100912777.1	hypothetical protein	Y
*P*. *spongiae* (NZ_CP023398.1)	SAO4_RS03165	WP_100912778.1	isoprenylcysteine carboxyl methyltransferase	Y
*Pseudoalteromonas donghaensis* HJ51 (NZ_CP032090.1)	D0907_RS13430	WP_016708241.1	isoprenylcysteine carboxylmethyltransferase	Y
*P*. *donghaensis* HJ51 (NZ_CP032090.1)	D0907_RS13435	WP_016708242.1	DUF2933 domain-containing protein	Y
*P*. *donghaensis* HJ51 (NZ_CP032090.1)	D0907_RS13440	WP_045979597.1	cupredoxin domain-containing protein	Y
*P*. *donghaensis* HJ51 (NZ_CP032090.1)	D0907_RS13445	WP_045979598.1	copper-translocating P-type ATPase	Y
*P*. *donghaensis* HJ51 (NZ_CP032090.1)	D0907_RS13450	WP_045979599.1	MerR family transcriptional regulator	Y
*P*. *donghaensis* HJ51 Plasmid (NZ_CP032091.1)	D0907_RS19240	WP_118845117.1	cupredoxin domain-containing protein	N*
*P*. *donghaensis* HJ51 Plasmid (NZ_CP032091.1)	D0907_RS19245	WP_118845119.1	copper-translocating P-type ATPase	N*
*Pseudoalteromonas nigrifaciens* KMM 661 (NZ_CP011036.1)	PNIG_RS05890	WP_004589265.1	MerR family transcriptional regulator	Y
*P*. *nigrifaciens* KMM 661 (NZ_CP011036.1)	PNIG_RS05895	WP_004589266.1	copper-translocating P-type ATPase	Y
*P*. *nigrifaciens* KMM 661 (NZ_CP011036.1)	PNIG_RS05900	WP_004589267.1	cupredoxin domain-containing protein	Y
*P*. *nigrifaciens* KMM 661 (NZ_CP011036.1)	PNIG_RS05905	WP_089367991.1	hypothetical protein	Y
*P*. *nigrifaciens* KMM 661 (NZ_CP011036.1)	PNIG_RS05910	WP_004589269.1	isoprenylcysteine carboxylmethyltransferase	Y
*Simiduia agarivorans* SA1 (NC_018868.3)	M5M_RS02810	WP_015045952.1	MerR family transcriptional regulator	Y
*S*. *agarivorans* SA1 (NC_018868.3)	M5M_RS02815	WP_037433085.1	copper-transporting ATPase	Y
*S*. *agarivorans* SA1 (NC_018868.3)	M5M_RS02820	WP_015045954.1	plastocyanin domain-containing protein	Y
*S*. *agarivorans* SA1 (NC_018868.3)	M5M_RS02825	WP_015045956.1	hypothetical protein	Y
*S*. *agarivorans* SA1 (NC_018868.3)	M5M_RS02830	WP_015045957.1	isoprenylcysteine carboxyl methyltransferase	Y
*Spongiibacter* sp. IMCC21906 (NZ_CP011477.1)	IMCC21906_RS14790	WP_008296010.1	isoprenylcysteine carboxyl methyltransferase	Y
*Spongiibacter* sp. IMCC21906 (NZ_CP011477.1)	IMCC21906_RS14820	WP_047013472.1	MerR family transcriptional regulator	Y
*Spongiibacter* sp. IMCC21906 (NZ_CP011477.1)	IMCC21906_RS14810	WP_040362992.1	copper-transporting ATPase	Y
*Spongiibacter* sp. IMCC21906 (NZ_CP011477.1)	IMCC21906_RS14805	WP_008284637.1	hypothetical protein	Y
*Salinimonas sediminis* strain N102 (NZ_CP031769.1)	D0Y50_RS10110	WP_117316793.1	DUF2933 domain-containing protein	Y
*S*. *sediminis* N102 (NZ_CP031769.1)	D0Y50_RS10115	WP_117318712.1	isoprenylcysteine carboxylmethyltransferase	Y
*S*. *sediminis* N102 (NZ_CP031769.1)	D0Y50_RS10210	WP_117316832.1	isoprenylcysteine carboxylmethyltransferase	Y
*S*. *sediminis* N102 (NZ_CP031769.1)	D0Y50_RS10220	WP_013755268.1	DUF2933 domain-containing protein	Y
*S*. *sediminis* N102 (NZ_CP031769.1)	D0Y50_RS10225	WP_044447459.1	cupredoxin domain-containing protein	Y
*S*. *sediminis* N102 (NZ_CP031769.1)	D0Y50_RS10230	WP_176582454.1	heavy metal translocating P-type ATPase	Y
*S*. *sediminis* N102 (NZ_CP031769.1)	D0Y50_RS10235	WP_117316834.1	MerR family transcriptional regulator	Y
*Salinimonas* sp. KX18D6 plasmid (NZ_CP039853.1)	FBQ74_RS17530	WP_117316834.1	MerR family DNA-binding protein	N*
*Salinimonas* sp. KX18D6 plasmid (NZ_CP039853.1)	FBQ74_RS17535	WP_117318713.1	copper-translocating P-type ATPase	N*
*Salinimonas* sp. KX18D6 plasmid (NZ_CP039853.1)	FBQ74_RS17540	WP_044447459.1	cupredoxin domain-containing protein	N*
*Salinimonas* sp. KX18D6 plasmid (NZ_CP039853.1)	FBQ74_RS17545	WP_013755268.1	DUF2933 domain-containing protein	N*
*Salinimonas* sp. KX18D6 plasmid (NZ_CP039853.1)	FBQ74_RS17550	WP_139758062.1	isoprenylcysteine carboxylmethyltransferase	N*
*Salinimonas* sp. KX18D6 plasmid (NZ_CP039853.1)	FBQ74_RS17645	WP_139758073.1	isoprenylcysteine carboxylmethyltransferase	N*
*Salinimonas* sp. KX18D6 plasmid (NZ_CP039853.1)	FBQ74_RS17650	WP_117316793.1	DUF2933 domain-containing protein	N*
*Salinimonas* sp. KX18D6 plasmid (NZ_CP039853.1)	FBQ74_RS17655	WP_139758074.1	cupredoxin domain-containing protein	N*
*Salinimonas* sp. KX18D6 plasmid (NZ_CP039853.1)	FBQ74_RS17660	WP_139758075.1	heavy metal translocating P-type ATPase	N*
*Cycloclasticus* sp. PY97N (NZ_CP023664.1)	CPC19_RS09700	WP_008296010.1	isoprenylcysteine carboxylmethyltransferase	Y
*Cycloclasticus* sp. PY97N (NZ_CP023664.1)	CPC19_RS09705	WP_022960189.1	DUF2933 domain-containing protein	Y
*Cycloclasticus* sp. PY97N (NZ_CP023664.1)	CPC19_RS09715	WP_096910763.1	cupredoxin domain-containing protein	Y
*Cycloclasticus* sp. PY97N (NZ_CP023664.1)	CPC19_RS09720	WP_096910813.1	Cu(2+)-exporting ATPase	Y
*Cycloclasticus* sp. PY97N (NZ_CP023664.1)	CPC19_RS09730	WP_096910765.1	MerR family DNA-binding transcriptional regulator	Y
*Oleispira antarctica* RB-8 (NZ_FO203512.1)	OLEAN_RS13750	WP_046009695.1	plastocyanin	N
*O*. *antarctica* RB-8 (NZ_FO203512.1)	OLEAN_RS13755	WP_046009696.1	copper-translocating P-type ATPase	N
*O*. *antarctica* RB-8 (NZ_FO203512.1)	OLEAN_RS19120	WP_046010563.1	isoprenylcysteine carboxyl methyltransferase	N, I
*O*. *antarctica* RB-8 (NZ_FO203512.1)	OLEAN_RS19125	WP_046010564.1	membrane protein	N
*O*. *antarctica* RB-8 (NZ_FO203512.1)	OLEAN_RS19130	WP_046010565.1	plastocyanin	N
*O*. *antarctica* RB-8 (NZ_FO203512.1)	OLEAN_RS19135	WP_084687646.1	copper-translocating P-type ATPase	N
*Thalassolituus oleivorans* R6-15 (NZ_CP006829.1)	R615_RS08695	WP_025265534.1	isoprenylcysteine carboxyl methyltransferase	N
*T*. *oleivorans* R6-15	R615_RS08700	WP_015486876.1	hypothetical protein	N
*T*. *oleivorans* R6-15	R615_RS08715	WP_015486873.1	hypothetical protein	Y
*T*. *oleivorans* R6-15	R615_RS08720	WP_051052465.1	copper-transporting ATPase	Y
*T*. *oleivorans* R6-15	R615_RS08725	WP_015486871.1	MerR family transcriptional regulator	Y
*Glaciecola nitratireducens* FR1064 (NC_016041.1)	GNIT_RS16210	WP_014110387.1	copper-transporting ATPase	N
*G*. *nitratireducens* FR1064 (NC_016041.1)	GNIT_RS16205	WP_014110386.1	cupredoxin domain-containing protein	N
*Idiomarina loihiensis* L2TR (NC_006512.1)	IL_RS06270	WP_011234469.1	cadmium-translocating P-type ATPase	N
*I*. *loihiensis* L2TR (NC_006512.1)	IL_RS06265	WP_011234468.1	cupredoxin domain-containing protein	N
*Oleiphilus messinensis* strain ME102 (NZ_CP021425.1)	OLMES_RS13415	WP_087464474.1	copper-translocating P-type ATPase	N
*Shewanella polaris* SM1901 (NZ_CP041036.1)	FH971_RS14175	WP_140234754.1	MerR family transcriptional regulator	Y
*S*. *polaris* SM1901 (NZ_CP041036.1)	FH971_RS14170	WP_140234753.1	copper-translocating P-type ATPase	Y
*S*. *polaris* SM1901 (NZ_CP041036.1)	FH971_RS14165	WP_037425971.1	cupredoxin domain-containing protein	Y

* indicates located on plasmid, I indicates flanked by site-specific integrase (OLEAN_RS19185)

## Results and discussion

### Copper growth assays

**Fig 1:**
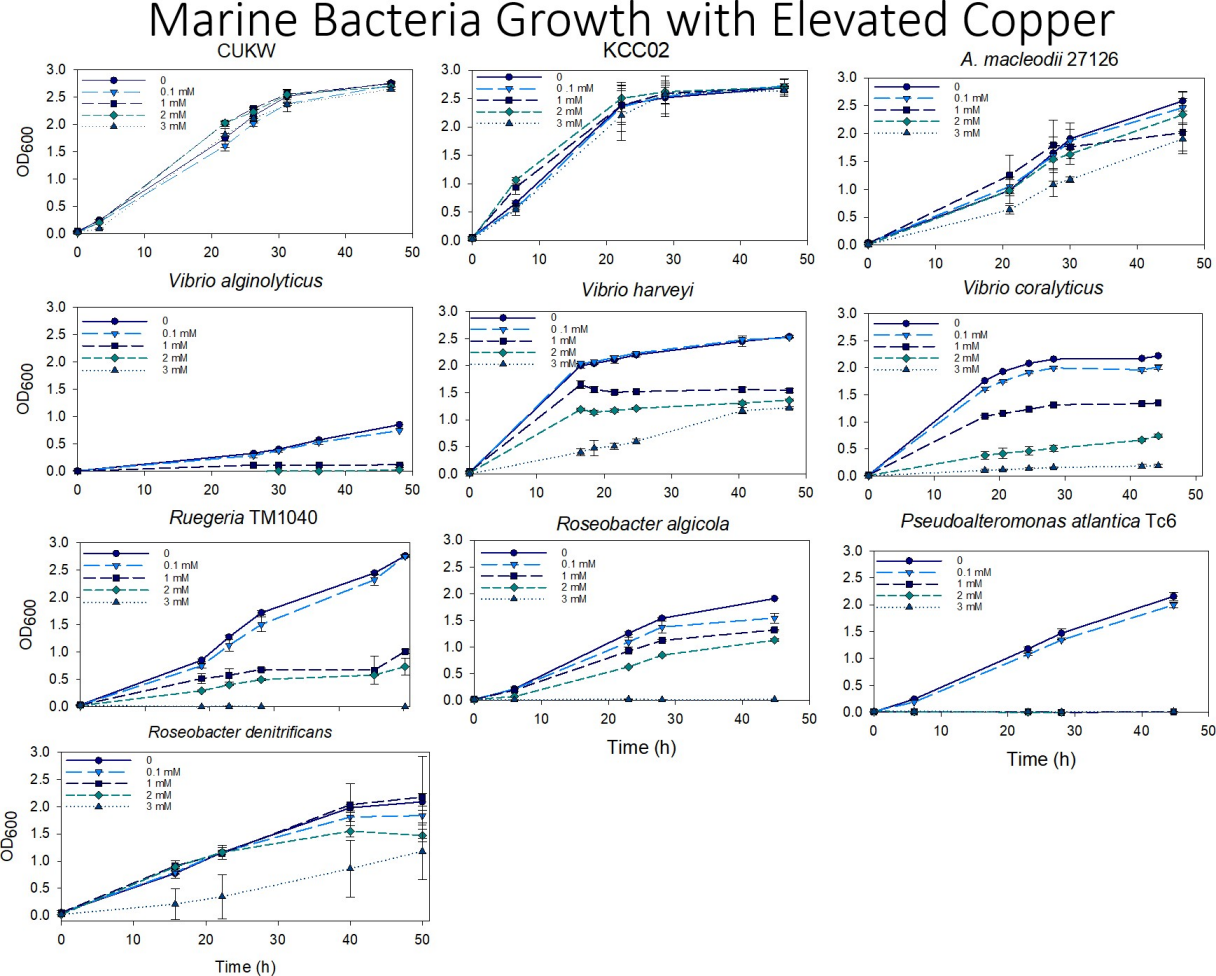
Marine bacteria copper resistance assays. Single colonies of marine bacterial species were inoculated into 3 ml of Burkholder’s B Formulation or Difco marine broth 2216 and grown overnight with agitation. Upon reaching late exponential phase, cultures were inoculated at a 1:100 dilution into 10 ml of fresh medium and copper added to final concentrations of 3 mM, 1 mM, 2 mM, or 100 μM. Cultures were incubated with agitation for ca. 48 h and growth recorded as the change in optical density at 600 nm over time. Error bars represent standard deviation of two biological replicates, with three technical replicates recorded for each.

Growth was not impaired at any copper concentration in either CUKW or KCC02; in both strains, growth at any copper concentration, including 3 mM, was nearly identical to that of the no-copper control (Fig 1). CUKW growth lagged slightly behind that of KCC02, with KCC02 entering stationary phase at ca. 22 h and CUKW at ca. 30 h. This is not surprising considering KCC02 was grown continuously in medium supplemented with 3 mM copper. *A*. *macleodii* 27126 achieved growth comparable to the no-copper control at all concentrations except 3 mM. At this concentration, the strain displayed impaired growth. In general, growth for all species in the presence of 100 μM copper was the same as for the no-copper control (Fig 1). *Vibrio alginolyticus* displayed reduced growth at 1, 2, and 3 mM copper, achieving a comparable level of growth at 2 and 3 mM at ca. 40 h. *V*. *coralyticus* displayed the general trend of decreased growth with increasing copper concentration, with little to no growth at 3 mM. *Rugeria* TM 1040 displayed reduced growth, at comparable levels, when grown with 1 or 2 mM copper, and no growth at 3 mM. *Roseobacter algicola* displayed reduced growth at 1 and 2 mM copper, with no growth at 3 mM. *Pseudoalteromonas atlantica* TC6 was not able to grow at any concentration other than 100 μM. *R*. *denitrificans* was able to grow at all concentrations, with growth at 1 and 2 mM comparable to the no-copper control. Overall, *A*. *macleodii* CUKW and KCC02 were the only species to display robust growth at 3 mM copper (Fig 1).

### Sequencing, assembly, and annotation

SMRT cell sequencing produced 81,437 subreads (831,750,960 bp) with an average length of 10,213 bp for CUKW and 131,535 subreads (1,239,730,565 bp) with an average read length of 9,425 bp for KCC02. A total of 63,509 (totaling 834,586,261 bp) reads was generated for CUKW, resulting in a 178-fold coverage. A total of 87,806 reads (totaling 1,246,350,428 bp) was generated for KCC02, resulting in a 256-fold coverage. The HGAP assembly output yielded 3 polished contigs for CUKW, indicating a circular chromosome of 4,647,095 bp and two plasmids, sized 212,603 bp and 178,065 bp. HGAP assembly yielded nine polished contigs for KCC02, indicating a circular chromosome of 4,642,293 bp, and three plasmids, sized 242,126 bp, 182,043 bp, and 258,061 bp (Table 6). Plasmid size, presence, and gene content was confirmed by pulsed field gel electrophoresis as recently reported [43]. The genome features of each strain are provided in Table 6. The Prokka pipeline was used for genome annotation; the resulting annotation was then used for genomic analysis, including sequence diversity, genome architecture, and gene content in CUKW and KCC02, as well as comparative genomic analyses among *Alteromonas* spp. for *copA* and *merR* variants. The CUKW and KCC02 genomes described in this study have been deposited in GenBank, BioProject PRJNA485824.

**Table 6:** Genome statistics of strains CUKW and KCC02.

	CUKW chromosome	pCUKW-212	pCUKW-178	KCC02 chromosome	pKCC02-243	pKCC02-180	pKCC02-258
**Total Length**	4,647,095	212,603	178,065	4,642,293	243,126	182,043	258,061
**GC content %**	44.7	40.8	42.4	44.7	43.3	46.6	42
**CDS**	3972	227	174	3967	230	217	268
**rRNA**	16	0	0	16	0	0	0
**tRNA**	71	0	0	71	0	0	0
**CRISPRs**	0	0	0	0	0	0	0

### Identification of copper-associated genes

In order to gain insights into the potential copper homeostasis networks of CUKW and KCC02, we compiled a database of Hidden Markov Models for copper-associated genes mapping to a manually curated set of proteins from the Cue, Cus, and Cop/Pco systems. Results are based on hits to these reference PFAM/TIGRFAM/COG models. Thus, the hits reported here are indicative of homology with the proteins used to make up the models. Hits were defined as sequences identified as mapping to the models with a maximum e-value cut-off of e^-30^. In CUKW, Cue system components as well several *cop*/*pco* copies are plasmid-based (pCUKW-178), while the majority of Cus homologs occur on the chromosome (S1 Table). This same trend occurred in KCC02 (S2 Table). CUKW possesses three copies of *copA*, two of which are located on the plasmid and one on the chromosome, and two *cueR*-like genes, both plasmid-based (S1 Table). There is a single hit to a CueO/PcoA, and it localizes to the plasmid. This is the same for KCC02, with two of three *copA*-like genes and both *cueR*-like genes located on the pKCC02-243 plasmid (the functional equivalent of pCUKW-178 [43], S2 Table) and a single plasmid-based CueO hit. Thus, except for the one chromosomal copy of *copA*, all Cue system elements are plasmid-based in both strains. Regarding the Cus system, most hits were most similar to CusA and CusB in both CUKW and KCC02. However, while many of the genes with hits to the efflux transporter CusB possess a domain similar to that of CusB, their specificity for copper remains to be experimentally verified. In keeping with previous comparative genomic analyses, in which *A*. *macleodii* genomes were found to be enriched in two-component systems and elements indicative of complex regulation and environmental sensing [44], CUKW and KCC02 contain a large number of genes associated with the two-component regulatory systems of Pco/Cop: of the 43 multisystem hits in CUKW, nearly all are similar to the sensor histidine kinase or response regulator of Pco/Cop. This same trend occurs in KCC02: of the 47 multisystem hits, all but two are most similar to the Pco/Cop two-component regulatory systems.

### Comparative genomics among marine species

A comparative genomics analysis was performed among CUWK and KCC02 and other marine species using the same methods and database as described for CUKW and KCC02. Hits were defined as sequences identified as mapping to the models with a maximum e-value cut-off of e^-30^. Marine species included many for which growth was assessed under a range of copper concentrations in this work (*A*. *macleodii* 27126; *Marinovum algicola*; *Pseudoalteromonas atlantica* TC6; *Roseobacter denitrificans*; *Rugeria* TM1040; *Vibrio alginolyticus*; *V*. *coralliilyticus*; *V*. *harveyi*) as well as *Marinovum algicola* and *A*. *macleodii* Balearic Sea. When a complete genome for the strain of interest was not available, multiple strains for the relevant species were used in the analysis (S3 Table). Results were grouped by copper-resistance category and reported as hits per strain and/or species.

**Fig 2:**
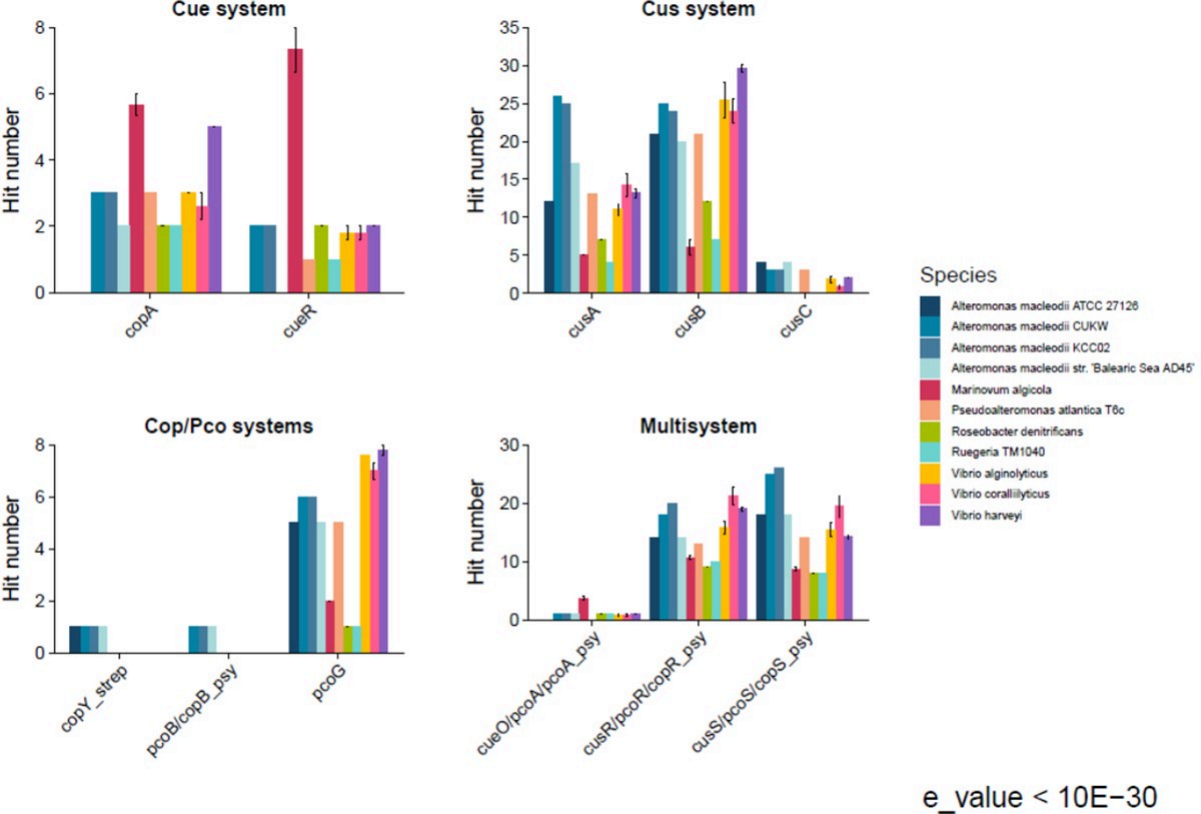
Number of hits to models of copper-associated proteins in CUKW and KCC02 in comparison to other marine bacterial species. Model names are shown on the X-axis. The number of hits per protein and per species were counted and grouped by the category of interest: Cue, Cus, Cop/Pco, and multisystem. An e-value of 10^−30^ served as the threshold for defining a hit.

Overall, *Alteromonas* possess a balanced enrichment of copper systems in comparison to other marine bacterial genera. While the analyses demonstrate that some species contain a greater number of counts for specific systems, *Alteromonas*, and in particular strains CUKW and KCC02, show consistent enrichment across all categories (Cue, Cus, Cop/Pco, Multisystem) (Fig 2). The number of CopA homologs in CUKW and KCC02 is comparable to most other species, with only *M*. *algicola* and *V*. *harveyi* containing more. *M*. *algicola* harbors the greatest number of CueR homologs, nearly four times as many as the other species analyzed here. This species also possessed the greatest number of hits to CueO (Fig 2). CUKW and KCC02 also possess a comparable number of CueR hits as for most of the other species (Fig 2).

Within the Cus system category, *Alteromonas* strains, in particular CUKW and KCC02, possess a greater number of putative CusA and CusB homologs than nearly all other species, with hits to CusB exceeded only by *V*. *alginolyticus* and *V*. *harveyi* (Fig 2). The Cus system is comprised of efflux pumps, and the specificity of the Cus components identified here (Cus A, B and C in addition to Cus R and CusS) remains to be experimentally verified, as CUKW and KCC02 display high resistance to other metals as well [43]. A general trend observed among most species was specificity of hits to either the Cue or the Cus system. For example, while *M*. *algicola* possessed the greatest number of hits to all elements of Cue, this species appears to possess a very limited Cus system. A similar pattern occurred in *Rugeria* TM1040 and *R*. *denitrificans*. The exception to this trend was *Alteromonas*, especially CUKW and KCC02, which both harbored substantial numbers of hits to both systems (Fig 2).

The Cop and Pco systems were combined here into a single category for analysis, since their components mapped to the same structural models. All species yielded at least one hit to PcoG, with a general trend of increased abundance in *Alteromonas* and *Vibrio* spp (Fig 2). *PcoG* is not part of the 7-member *pco* operon, but instead occurs (along with *copF*) on a transposable element-flanked island of 19 genes that includes adjacent *cus* and *pco* clusters known as Copper Homeostasis and Silver Resistance Island (CHASRI) in multiple bacterial species [42]. *Alteromonas* was the only genus which yielded hits to PcoB/CopB as well as the CopY of *Streptococcus* (Fig 2). In *Streptococcus*, CopY functions as a repressor of the *cop* operon [45, 46]. Recent work examining the influence of metal crosstalk on CopY activation and function demonstrated novel mechanisms for copper processing within the pneumococcal system [46]. While the role of this putative CopY homolog in *A*. *macleodii* remains to be experimentally verified, its presence hints at the presence of a diverse regulatory network related to metal homeostasis within this species.

The multisystem category encompasses models that map to proteins in more than one system. This category contained three elements: CueO/PcoA/CopA (“CueO”); CusR/PcoR/CopR (“CusR”); and CusS/PcoS/CopS (“CusS”). CueO is a multicopper oxidase that may function to protect periplasmic enzymes from copper-induced damage [8]. CueO was identified in all species except *A*. *macleodii* 27126 and *P*. *atlantica*. It occurred as a single hit in most species, including *Alteromonas* CUKW and KCC02 (Fig 2). Homologs of the Cus two-component regulatory system, the histidine kinase CusR and response regulator CusS, were identified in all species (Fig 2). The greatest hits to CusR occurred in CUKW and KCC02 as well as *V*. *coralliilyticus* and *V*. *harveyi* (Fig 2). An even greater number of CusS hits were identified in CUKW and KCC02, with nearly 30 hits identified in both strains (S1 and S2 Tables, Fig 2).

When assimilating bioinformatic analysis with marine bacterial species growth data, the following trends were noted and are couched primarily in comparison with CUKW and KCC02. In *V*. *harveyi*, our analyses identified a greater number of hits to CopA than all but *M*. *algicola*, reduced CusA and CusS but high abundance of CusB hits, and an absence of hits to Cop/Pco elements. *V coralyticus* displayed a slight reduction in CopA hits, reduced CusA and CusC hits, and lacked hits to elements of the Cop/Pco system. *Rugeria* TM 1040 showed reduced levels of Cue and Cus system elements in comparison to CUKW and KCC02 while also lacking elements of the Cop/Pco system. *Pseudoalteromonas atlantica* TC6 showed reduced CueR and CusA hits, and an absence of Cop/Pco elements. *Roseobacter denitrificans* showed a reduced number of hits for all but CueR in comparison to CUKW and KCC02. Collectively, the growth data corroborate the comparative genomic analyses that revealed *Alteromonas* are enriched in homologs of known copper resistance proteins derived from the *E*. *coli* and *Pseudomonas* model systems. However, these analyses do not preclude other, potentially novel, copper resistance mechanisms used by the marine species addressed here, as exemplified by *R*. *denitrificans*, which displayed a reduced number of hits to the model *E*. *coli* and *Pseudomonas* systems yet was able to grow at all copper concentrations, albeit with reduced growth at 3 mM.

### Comparative genomics with model species for copper resistance

A comparative analysis was also performed between CUKW and KCC02 and reference strains associated with high copper tolerance phenotypes or those used in genetic analyses to elucidate copper resistance genes (Table 7) using the same criteria as described above for marine genera. Environment has been particularly influential in elucidating the mechanisms of copper tolerance to date, as the main models of copper tolerance were originally identified in species isolated from copper-rich environments (i.e. *E*. *coli* [47, 48], *Salmonella* [49], and *Enterococcus* [50] from pigs fed copper-rich diets, *P*. *syringae* [21] and *Xanthomonas* sp. [51] from plants treated with copper). The presence and abundance of copper in coastal marine systems is increasing due to its use as an algaecide and as an anti-fouling (AF) agent on ship hulls [24, 25]. CUKW was isolated from copper coupons, with copper in the form of cuprous oxide (Cu_2_O), being tested for use in marine vessel coatings. Dissolved oxygen in seawater oxidizes Cu^1+^ complexes to Cu^2+^, which serves as the main biocidal ion [25]. The typical copper concentrations in coastal seawater are *ca*. 12.7 nM (total dissolved copper), with a free cupric ion concentration, [Cu^2+^] of 10^−10^ mM [52]. This is significantly lower than that encountered by microbes on a copper oxide coating (approx. 6 mM in a 10 cm^2^ area at a loading of 0.1% (w/w)).

**Table 7:** Model species or those from copper-rich environments used in analysis.

Strain	Isolation Source/Genetic Study	Reference
*Xanthomonas euvesicatoria* (formerly perforans) LH3	tomato; copper-resistant phenotype; plasmid-based	[51]
*Pseudomonas syringae* pv tomato PT23	identification of cop genes	[21]
*Xanthomonas citri* Xc-03-1638-1-1	grapefruit; copper-tolerant phenotype; plasmid-based	[53]
*Xanthomonas vesicatoria* LMG911	tomato; copper-resistant phenotype; plasmid-based	[51]
*Xanthomonas euvesicatoria* LMG930	pepper; copper-resistant phenotype; plasmid-based	[51]
*Xanthomonas gardneri* JS749-3	tomato; copper-resistant phenotype;plasmid-based	[51]
*Xanthomonas gardneri* ICMP7383	tomato; copper-resistant phenotype; plasmid-based	[51]
*Xanthomonas vesicatoria* LM159	pepper; copper-resistant phenotype; plasmid-based	[51]
*Salmonella typhimurium* S7	copper-fed pigs; chromosome-based	[49]
*Citrobacter freundii* NCTC9750	isolation source not available (NZ_LR134118)	
*Escherichia coli* DH5alpha	studies on copper two-component system	[13]
*Escherichia coli* KSC64	copper-fed pigs; plasmid-based (pco)	[47]
*Escherichia coli* KSC9	copper-fed pigs; plasmid-based (pco)	[47]
*Escherichia coli* 77 3009 5	copper-fed pigs; chromosome+mobile island	[48]
*Escherichia coli* KSC207	copper-fed pigs; plasmid-based (pco)	[47]
*Escherichia coli* KSC1031	copper-fed pigs; plasmid-based (pco)	[47]
*Escherichia coli* W3110	AP009048.1; genetic studies on Cue and Cus	[8, 16]
*Enterococcus faecium* A17sv1	copper-fed pigs; copper-resistant phenotype; plasmid-based tcrB (transferable copper resistance)	[50]

**Fig 3:**
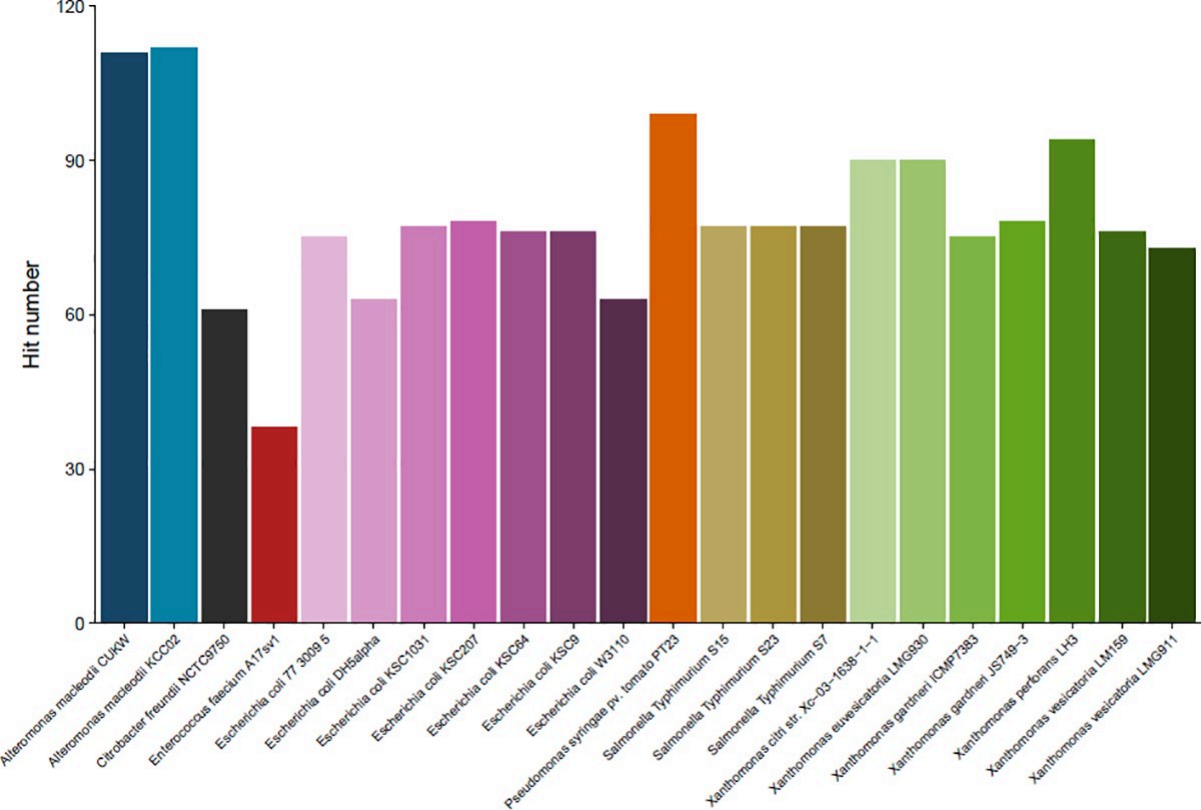
Total number of hits across all systems for copper-associated proteins in CUKW and KCC02 in comparison to bacterial species isolated from copper-rich environments. Model names (Table 7) are shown on the x-axis. An e-value of 1^−30^ served as the threshold for defining a hit.

**Fig 4:**
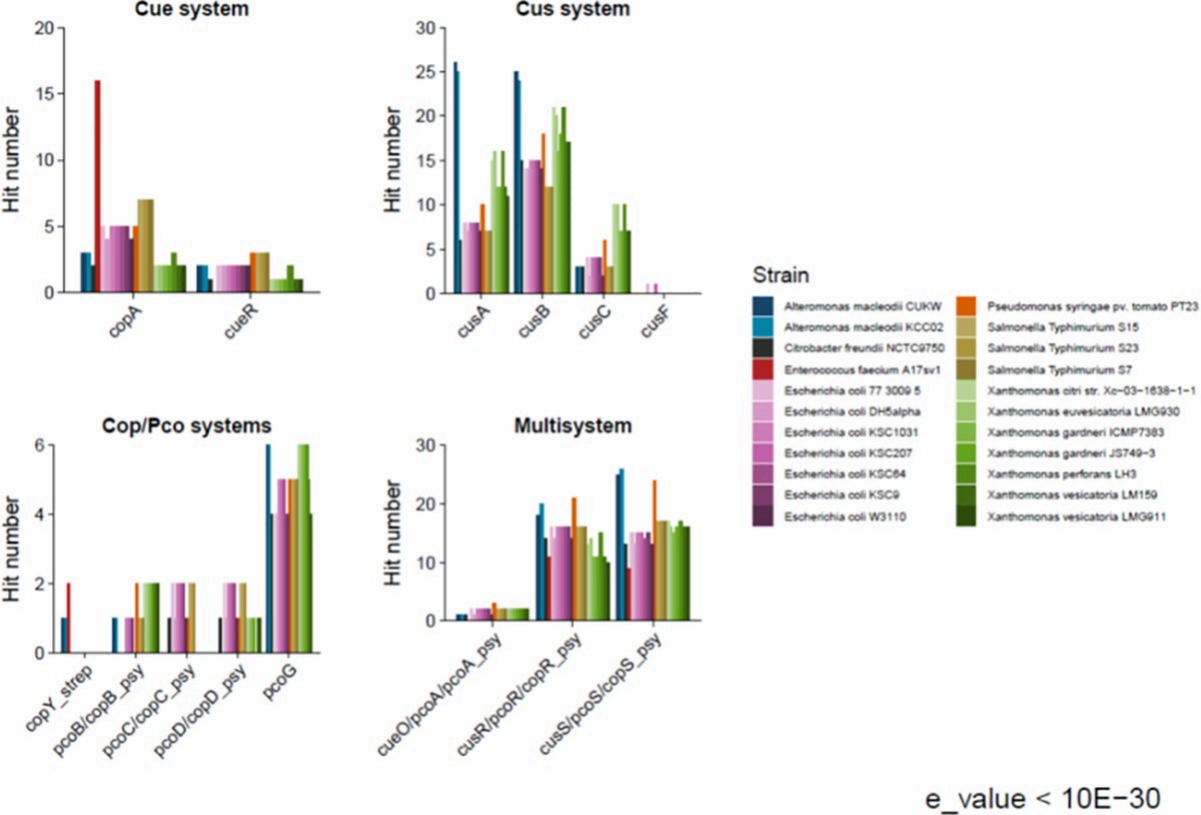
Number of hits to models of copper-associated proteins in CUKW and KCC02 in comparison to bacterial species isolated from copper-rich environments. Model names (Table 7) are shown on the X-axis. The number of hits per protein and per species were counted and grouped by the category of interest: Cue, Cus, Cop/Pco, and Multisystem. An e-value of 1^−30^ served as the threshold for defining a hit.

Even when compared to these copper-associated reference strains, the genomes of *Alteromonas* strains CUKW and KCC02 showed consistent enrichment in copper-associated genes, with only *Pseudomonas syringae* pv. tomato PT23 (the isolation strain of the CopABCD system) showing similar levels of consistent enrichment (Fig 3). When considering the cumulation of all systems, the number of hits to known copper homologs in CUKW and KCC02 (110 and 112, respectively) is greater than that of all other species, including *P*. *syringae* (99) (Fig 3, S4 Table). Overall, the trends recorded when comparing CUKW and KCC02 copper-associated genetic elements to other marine species also occurred when comparing to species with copper-resistant phenotypes. Within the Cue system, a comparable number of hits was obtained in both CUKW and KCC02 as for most other species isolated from copper-associated environments (Fig 4, S4 Table). CUKW and KCC02 possess greater hits to Cus system elements than other species, even greater than those recorded for *Xanthomonas* sp. (Fig 4). Cus models are derived from RND transporters; this abundance of hits to Cus-derived systems suggests efflux may be a primary mechanism by which CUKW and KCC02 overcome copper challenges. The specificity of these putative efflux pumps remains to be verified, as CUKW and KCC02 are also highly tolerant of other metals as well [43]. A very high abundance of hits to putative homologs of the Cus two-component regulatory system was recorded for both CUKW and KCC02. For both CusR and CusS, CUKW and KCC02 possess a greater number of hits than nearly all other species, with only *P*. *syringae* having a comparable number of hits (Fig 4). Within the Cop/Pco system, the greatest number of hits to PcoG was found to occur in *Alteromonas* and *Xanthomonas* (Fig 4). These collective comparative genomic analyses indicate that CUKW and KCC02 are consistently enriched in copper systems in comparison to other common marine species as well as copper-associated reference strains (Figs 3 and 4), and include elements not identified in the other species (i.e. CopY, PcoB). The large number of potential Cus regulators, combined with the identification of the CopY homolog, indicates a complex regulatory network within these strains for environmental sensing and response.

It is worth noting that data were analyzed with a range of e-values. Increasing or decreasing the stringency threshold produced the same results: *Alteromonas*, and in particular CUKW and KCC02, showed consistent enrichment across systems regardless of e-value (S1–S6 Figs). The consistency of these results across a wide range of e-values indicates that *Alteromonas* strains, and in particular CUKW and KCC02, possess a wider repertoire of copper-associated genes, consistent with their ability to grow at high copper concentrations and their isolation habitat. Of particular note is the fact that increasing the e-value threshold criterion from e^-30^ to e^-10^ identified putative additional elements of the Pco/Cop system in CUKW and KCC02, including CopZ, PcoB/CopB, PcoC/CopC, and PcoD/CopD as well as an additional seven CueR homologs in both (S1 Fig, S5 and S6 Tables). Homology results at e-values higher than e^-30^ must be interpreted with caution, since the ability of reference models to discern copper-specific binding pockets in P-type ATPases, transcriptional regulators and other copper-associated proteins decreases with sequence divergence. Nonetheless, hits at the e^-10^ cut-off likely represent bona fide metal-associated proteins, and their over-abundance in copper-tolerant *Alteromonas* strains may be partly due to overlapping substrate specificities that can be co-opted for copper-homeostasis at high copper concentrations [54]. These comparative genomic analyses demonstrating the enrichment in homologs of known copper resistance proteins derived from the *E*. *coli* and *Pseudomonas* model systems corroborate the growth of CUKW and KCC02 at copper levels inhibitory or lethal to other marine species. In agreement with the physiology data, both CUKW and KCC02 possess a diverse genetic repertoire of homologs associated with copper resistance in other species.

### Copper-induced expression of plasmid-based copper genes

**Fig 5:**
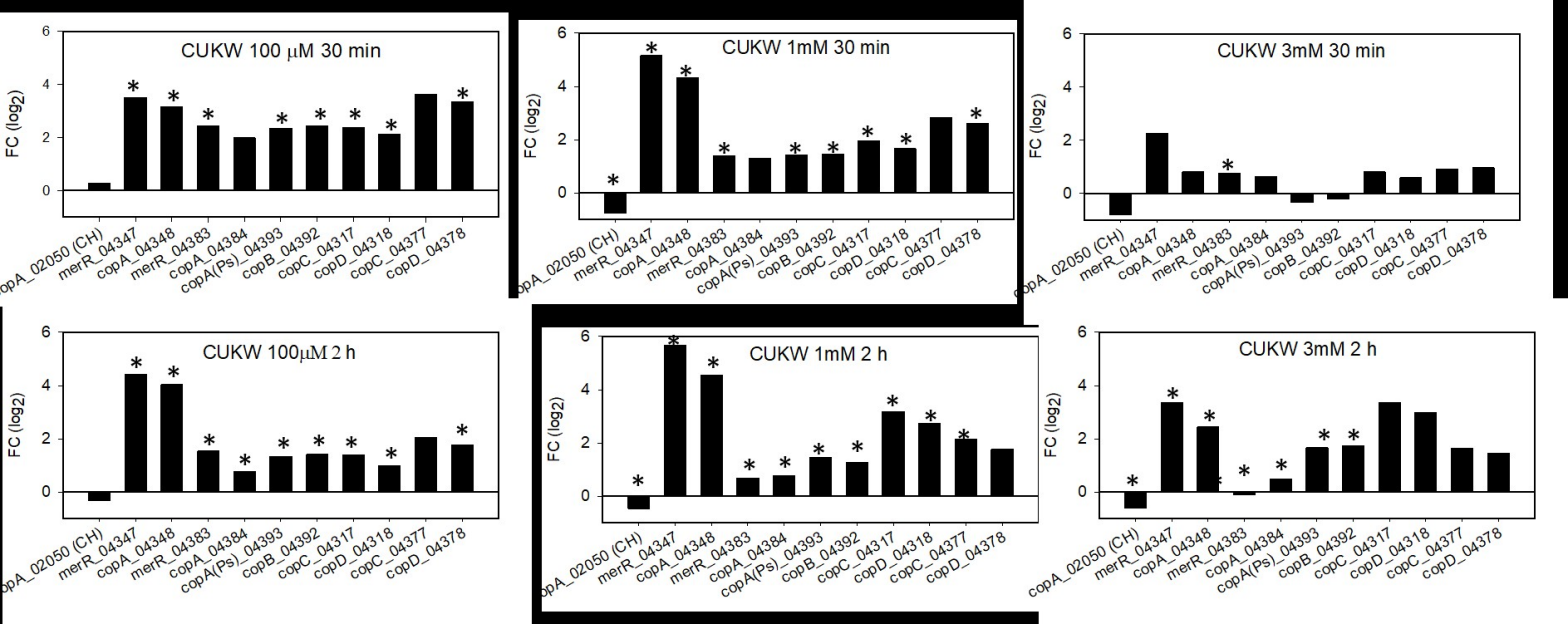
CUKW expression profiling with subset of copper-associated homologs from *E*. *coli* Cue and *Pseudomonas* Cop systems under a range of copper concentrations. Overnight cultures were diluted 1:100 into Burkholder’s B medium and incubated for 6 h, whereupon copper was added to final concentrations of 100 μM, 1mM, and 3mM. Samples were collected at 30 min and 2 h for expression profiling. copA(Ps) = designates *copA* of *P*. *syringae* system, of which *copB*, *C*, and *D* also belong. CH indicates gene is located on the CUKW chromosome. Gene expression was calculated using the ΔΔC_T_ method, with expression normalized to the reference gene *pfk*. Fold-change presented as log_2_-transformed values. * indicates significant difference between treatment (100 μM, 1 mM, or 3 mM copper, as designated in each panel) and control (no copper) at 30 min and 2 h (p < 0.05).

**Fig 6:**
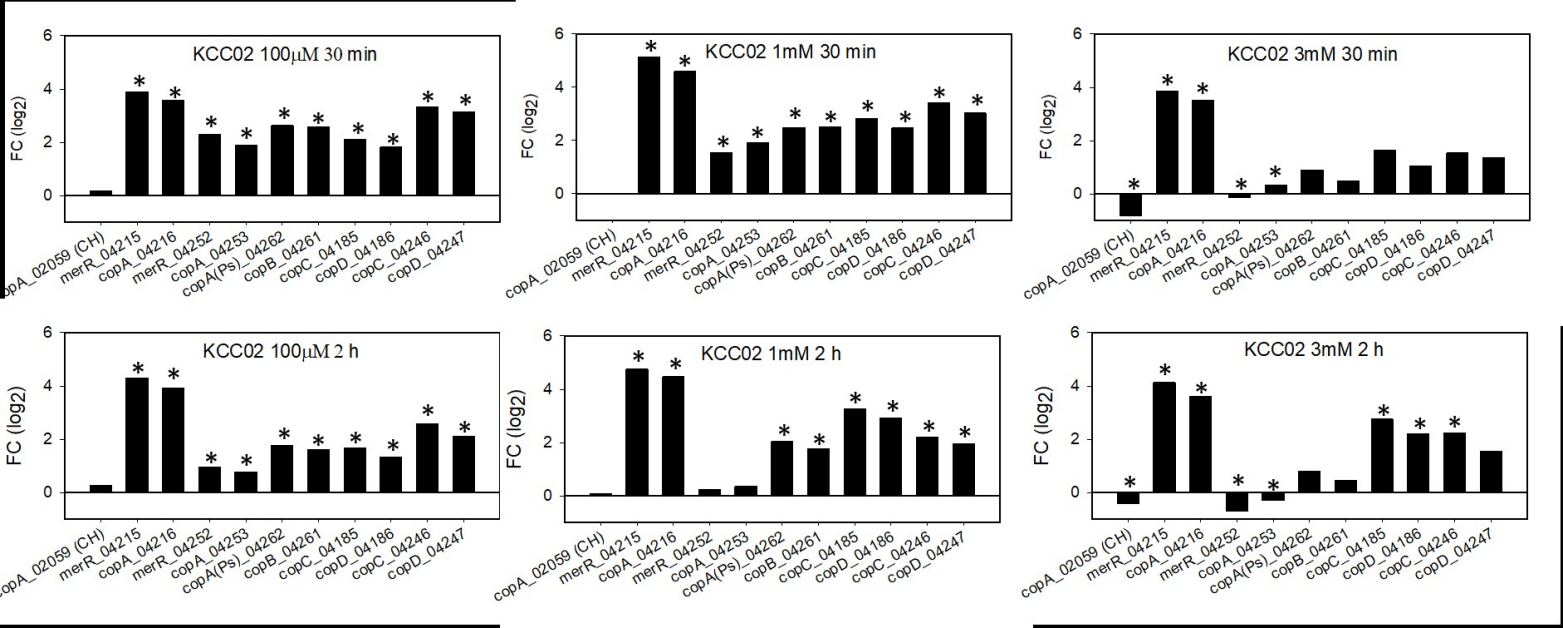
KCC02 expression profiling with subset of copper-associated homologs from *E*. *coli* Cue and *Pseudomonas* Cop systems under a range of copper concentrations. Overnight cultures were diluted 1:100 into Burkholder’s B medium and incubated for 6 h, whereupon copper was added to final concentrations of 100 μM, 1mM, and 3mM. Samples were collected at 30 min and 2 h for expression profiling. copA(Ps) = designates *copA* of *P*. *syringae* system, of which *copB*, *C*, and *D* also belong. CH indicates gene is located on the CUKW chromosome. Genes are presented in the same order as for Fig 5 (CUKW) and are homologous to those in CUKW but are referred to by their loci. Refer to Table 3 for corresponding CUKW-KCC02 loci names. Gene expression was calculated using the ΔΔC_T_ method, with expression normalized to the reference gene *pfk*. Fold-change presented as log_2_-transformed values. * indicates significant difference between treatment (100 μM, 1 mM, or 3 mM copper, as designated in each panel) and control (no copper) at 30 min and 2 h (p < 0.05).

The findings of three putative CopA homologs, one chromosomal and two plasmid-based, led us to examine which, if any, were induced upon exposure to copper. Expression profiling of all three revealed that the plasmid-based variants were induced at high copper concentrations in both CUKW (Fig 5) and KCC02 (Fig 6), while the chromosomal copy (CUKW_02050) was repressed or showed no change in expression (Figs 5 and 6).

The identification of two nearly identical *copA* clusters, each containing a gene encoding a MerR-family transcriptional regulator (putative *cueR*) immediately upstream of *copA*, led us to examine whether both *copA* variants were induced, as well as the potential role of the two *merR* variants in the regulation of both *copA* genes. In both CUKW and KCC02, the one plasmid-based *merR*-*copA* (CUKW_04348, hereafter referred to as the first variant) was consistently highly induced at 100 μM and 1 mM copper, much greater than that recorded for the second *copA* variant (CUKW_04384). Expression of this first *copA* variant decreased substantially (<2-fold) in CUWK upon exposure to 3 mM copper; while expression in KCC02 (KCC03_04216) was comparable to the level recorded for 100 μM (ca. 12-fold linear). As growth was delayed but not inhibited at 3 mM copper in CUKW (Fig 1), this difference in expression suggests alternative pathways may be used in the immediate response to this copper challenge. Expression of the first *merR* variant (CUKW_04247) mirrored that of the first *copA* variant, with high levels of induction at 100 μM and 1 mM and increasing over time at 3 mM copper. The second *copA* variant (CUKW_04384) was also induced at 100 μM and 1 mM, though not to the levels of the first variant; regulation of the second *merR* variant mirrored that of its *copA*. The correlation in expression levels between the two *merR* variants with their respective *copA* variants strongly suggest that each *copA* variant is independently regulated by its own *merR* rather than one *merR* controlling both *copA* variants. MerR family regulators operate typically as transcriptional activators and in several systems, such as the *P*. *putida cueR*, they are co-transcribed with their target genes, leading to correlation in gene expression levels [55]. No reports exist in the literature to our knowledge that describe this scenario of tandem *merR(CueR)-copA* copies and how they are regulated. In general, the active CueR binds two copper ions in its metal-binding domain, and upon binding induces expression of *copA* [56, 57]. CopA is regulated by CueR and has been shown to respond in a linear fashion to copper concentrations (albeit lower levels than those employed here) [56, 58].

The mega-plasmids of both CUKW and KCC02 harbor copper resistance genes belonging to two different reference systems (*E*. *coli* Cue, *P*. *syringae* Cop), prompting us to examine whether one or both systems were induced. Increasing evidence indicates that the periplasmic binding protein CopC frequently occurs with only the inner membrane protein CopD, typically as a fusion protein [59]. Genomic analysis indicates that this occurs in *Alteromonas*. One set of *copC* and *copD* genes, *copC* (CUKW_04377) and *copD* (CUKW_04378), respectively, with no apparent *copA* or *copB* homologs in the vicinity, was induced 10- to 13-fold at both 100 μM and 1mM (Figs 5 and 6). However, expression decreased to ca. 2-3-fold in both strains at 3 mM copper. Following 2 h exposure, the expression of another set of *copC* and *copD*-like genes, (CUKW_04317,CUKW_04318), respectively, increased to levels greater than the set at 30 min. Additionally, at 3 mM, this second set was expressed at levels comparable to those recorded for 1 mM copper, unlike other copper-associated genes whose expression decreased or was repressed at 3 mM copper (Figs 5 and 6). Collectively, these data indicate that CUKW and KCC02 respond to copper challenges through activation of multiple copper systems, with combined genomic and phylogenetic data suggesting a flexible genome that enabled the acquisition and re-arrangement of some of these systems. In both CUKW and KCC02, in addition to the two putative *merR*-like (i.e. putative *cueR*) genes located immediately upstream to the two plasmid-based *copA* genes, there are an additional three putative *cueR* genes situated throughout the plasmids. The high number of transcriptional regulators incorporated in their genomes suggests that different two-component systems may be utilized depending on copper level and growth phase.

### Characterization and phylogenetic distribution of *copA* variants

**Fig 7:**
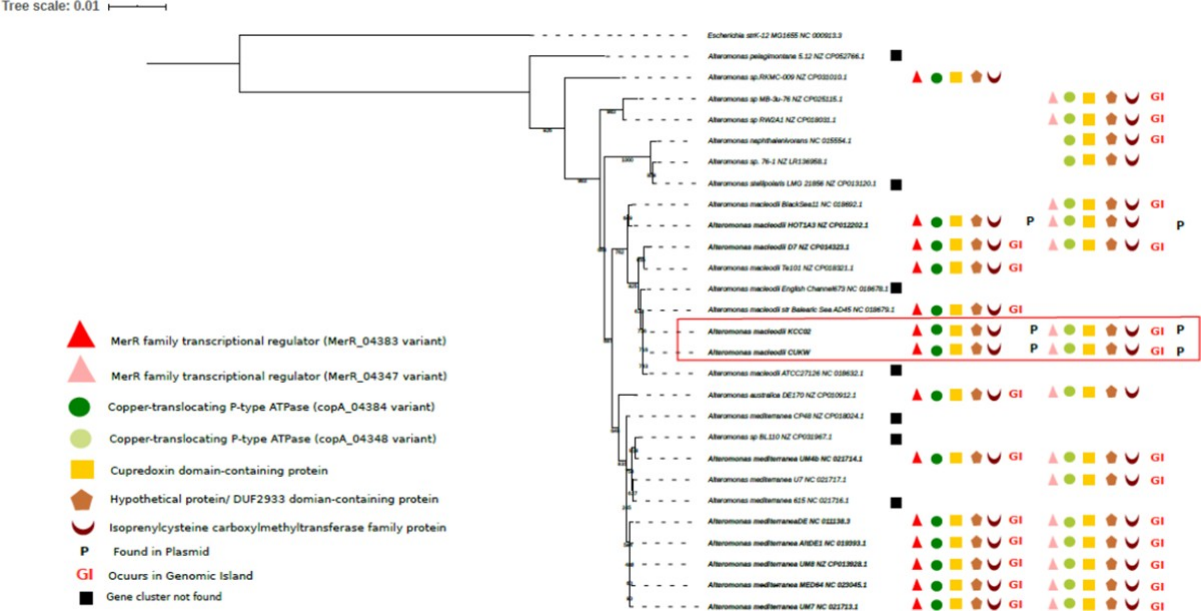
MerR-copA variant distribution among *Alteromonas*. Phylogenetic analysis of 16S ribosomal RNA sequence was done with all complete genomes of *A*. *macleodii* and *A*. *mediterranea* including a representative species from other *Alteromonas* genera available on NCBI RefSeq database. *Escherichia coli* was used as an outgroup to root the tree. The nucleotide sequences for 16S rRNA were downloaded from the NCBI database. Multiple sequence alignments of the sequences were performed using ClustalX and poorly aligned positions were filtered using Gblocks1. Phylogenetic trees were constructed using a bootstrap neighbor joining algorithm. The *copA* variants and surrounding gene clusters were mapped onto the phylogenetic distribution of 16S ribosomal RNA phylogeny using Inkscape editor. The two variants are differentiated by color. The two *merR* variants (MerR_04383 and MerR_04347) are shown as red and pink triangles, respectively. The two *copA* variants (CopA_04384 and CopA_04348) are shown as dark green and light green circles, respectively. “P” indicates the presence of the gene cluster on a plasmid and “GI” indicates the gene cluster occurs within a genomic island. The gene cluster found in *A*. *macleodii* strains CUKW and KCCO2 are highlighted in red.

As the sequence and genome arrangement of the plasmid-based *copA* variants are identical in CUKW and KCC02, only CUKW is described here. The two CUKW CopA variants are 71% similar; the primary difference in amino acid composition is a segment of 35 amino acids at the start of CUKW_04348. The gene content and organization surrounding the two *copA* variants on pCUKW-178 is identical: a MerR-like transcriptional regulator is situated immediately upstream of *copA*, followed by genes putatively encoding a cupredoxin-domain containing protein, a conserved hypothetical protein, and isoprenylcysteine carboxylmethyltransferase (Table 8). Intriguingly, the first *merR*-*copA* gene cluster occurs within a predicted GI on the pCUKW-178 (Fig 7, S7 Table).

**Table 8:** CUKW loci IDs for duplicated *copA* gene cluster.

Locus ID	Locus ID	Description
Amac_CUKW_04347	Amac_CUKW_04383	MerR family transcriptional regulator
Amac_CUKW_04348	Amac_CUKW_04384	Copper-translocating P-type ATPase
Amac_CUKW_04349	Amac_CUKW_04385	Cupredoxin-domain containing protein
Amac_CUKW_04350	Amac_CUKW_04386	hypothetical protein
Amac_CUKW_04351	Amac_CUKW_04387	Isoprenylcysteine carboxylmethyltransferase

The two *merR* variants also occur within the respective *A*. *mediterranea* and *A*. *macleodii* genomes that harbor either or both of the *copA* variants. The two putative MerR family transcriptional regulators share 67% similarity. Both are classified under the conserved protein domain family cd04787. In general, most of the MerR family regulators are triggered by environmental stimuli, with a subgroup of the family specific to metal ions, though the mechanisms of metal differentiation remain unclear [60]. This subgroup, which includes MerR itself, is proposed to have evolved to generate a variety of specific metal‐responsive regulators via fine‐tuning the sites of metal recognition [60].

The presence and sequence identity of the two plasmid-based *copA* homologs in the CUKW and KCC02 genomes is more similar to that found within *A*. *mediterranea* genomes than *A*. *macleodii*. Of the fully sequenced *A*. *macleodii* genomes, only two possess two *copA* variants: D7 and HOT1A3 (Fig 7, Table 4). In HOT1A3, as with CUKW and KCC02, both copies are located on a plasmid. D7 does not possess plasmids; both copies occur on the chromosome. *A*. *macleodii* Balearic Sea AD45 and Te101 possess a copy of the 04384 variant; the genome of strain Black Sea contains a copy of the 04348 variant. Both variants are found on the genomes of *A*. *mediterranea* strains DE, U10, UM8, UM4B, UM7, MED64, and AltDE1. Other *A*. *macleodii* strains for which closed genomes are available: 27126 and English Channel 673, do not possess orthologs of either variant (Fig 7). The arrangement of *copA* variants hence varies widely within *Alteromonas* and is quite distinct in CUKW and KCC02 from closely related *A*. *macleodii* strains.

**Fig 8:**
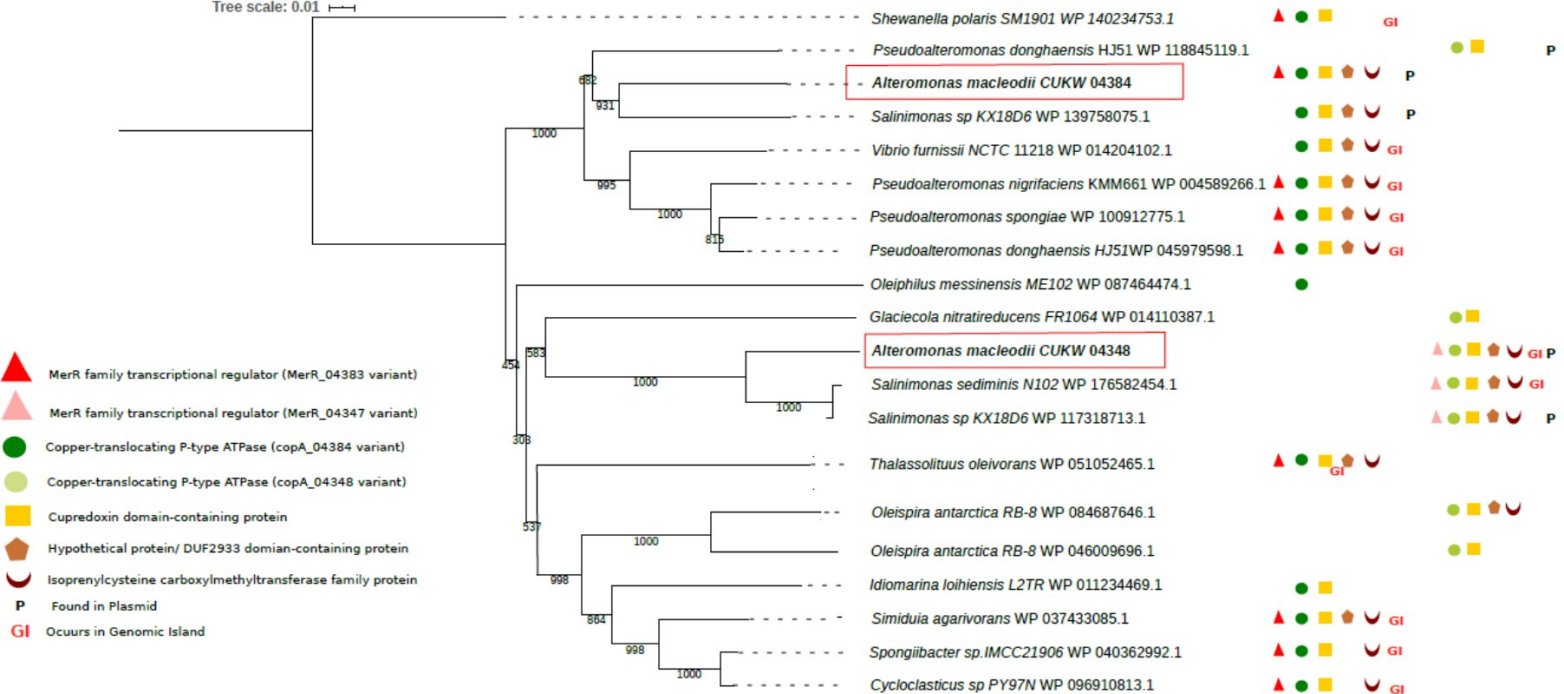
Distribution of CopA and MerR variants among bacterial genera. Phylogenetic analysis of the copper-translocating P-type ATPase (CopA) was done with bacterial species showing at least 45% sequence similarity with either CopA variant of *A*. *macleodii* CUKW. Only species with complete genome sequences available at NCBI RefSeq database were included. The amino acid sequences for CopA were downloaded from the NCBI database. Multiple sequence alignments of the sequences were performed using ClustalX and phylogenetic trees were constructed using a bootstrap neighbor-joining algorithm. The specific gene clusters surrounding the *copA* region in those species were identified by protein blast analysis against ones from *A*. *macleodii* CUKW. The *copA* variants and surrounding gene clusters were mapped onto the phylogenetic tree using Inkscape editor. The two variants are differentiated by color. The two *merR* variants (MerR_04383 and MerR_04347) are shown as red and pink triangles, respectively. The two *copA* variants (CopA_04384 and CopA_04348) are shown as dark green and light green circles, respectively. “P” indicates the presence of the gene cluster on a plasmid and “GI” indicates the gene cluster occurs within a genomic island. The two *copA* variants of *A*. *macleodii* CUKW are highlighted in red rectangular boxes.

The results of the phylogenetic analysis based on CopA sequences demonstrate that the two *copA* variants found in CUKW group into two distinct clusters (Fig 8). CUWK_04348 is part of cluster that consists of species that include the genera *Paraglaciecola*, *Aliiglaciecola*, *Idiomarina*, and *Simiduia*; while CUKW_04384 groups into a cluster dominated by *Pseudoalteromonas*. The results of IslandViewer and comparative genome analyses revealed that *copA* occurs on a predicted GI across multiple species and that, in many, it includes the “core” gene cluster identified in CUKW comprised of *merR*, *copA*, and genes coding for a cupredoxin domain-containing protein and an isoprenylcysteine methyltransferase, albeit with slight variations (Fig 8, Table 5).

One of the copies of this cluster (containing the CUKW_04348 copA variant) also occurs on a GI within the CUKW plasmid pCUKW-178 (Fig 8, S7 Table). In addition to the *copA* cluster (*merR*, *copA*, and genes coding for a hypothetical protein, cupredoxin domain-containing protein, and an isoprenylcysteine methyltransferase) the GI contains hydrogenase genes, genes for hypothetical proteins, a TolC family protein, and a heavy metal transport gene in addition to several transposases (S7 Table). No elements indicative of mobility were found within the GI.

While the means by which CUKW (and KCC02) originally acquired and why they maintain this collection of resistance genes is not fully known, it may, at least in part, be explained by their lifestyle and the genome attributes of the genus in general. *Alteromonas* is an *r*-selected specialist whose fitness success is derived from its ability to exploit transient niches. Part of its success may be accounted for by its flexible genome. *Alteromonas* as a genus displays substantial genome plasticity. Comparative genomic analysis of *Alteromonas* species and strains from varied environments has identified conserved core and flexible genome regions, and analysis of plasmids and conjugative elements has revealed a modular and dynamic framework that drives gene flux throughout the genus. It has led to a proposed model in which clones diverge, forming different clonal lineages, and that the flexible genome that defines the different isolates mainly occurs in flexible genomic islands [61, 62]. The flexible genome consists of multiple genomic islands and gene cassettes that can also occur on plasmids and integrative conjugative elements, indicating these mobile elements may serve as vectors for the transfer of these genomic islands, especially within strains and/or clonal populations [61, 63].

Twelve GI’s were identified via Island Viewer on the CUKW chromosome (S8 Table, S7 Fig) (As the chromosome content is very similar between CUKW and KCC02, only CUKW is discussed here.) Of these, one harbored genes coding for two CusA/CzcA family heavy metal efflux RND transporters (CUKW_00254 and CUKW_00255) with integrases (also within the GI) situated in the nearby genomic vicinity of a second that contained two genes coding for RND transporters (CUKW_01880 and CUKW_01881). Both GIs also contained multiple other genes and thus did not appear exclusive for copper resistance genes. Analysis of the genomic neighborhoods of chromosome-based copper resistance genes identified very limited presence of mobile genetic elements.

**Fig 9:**
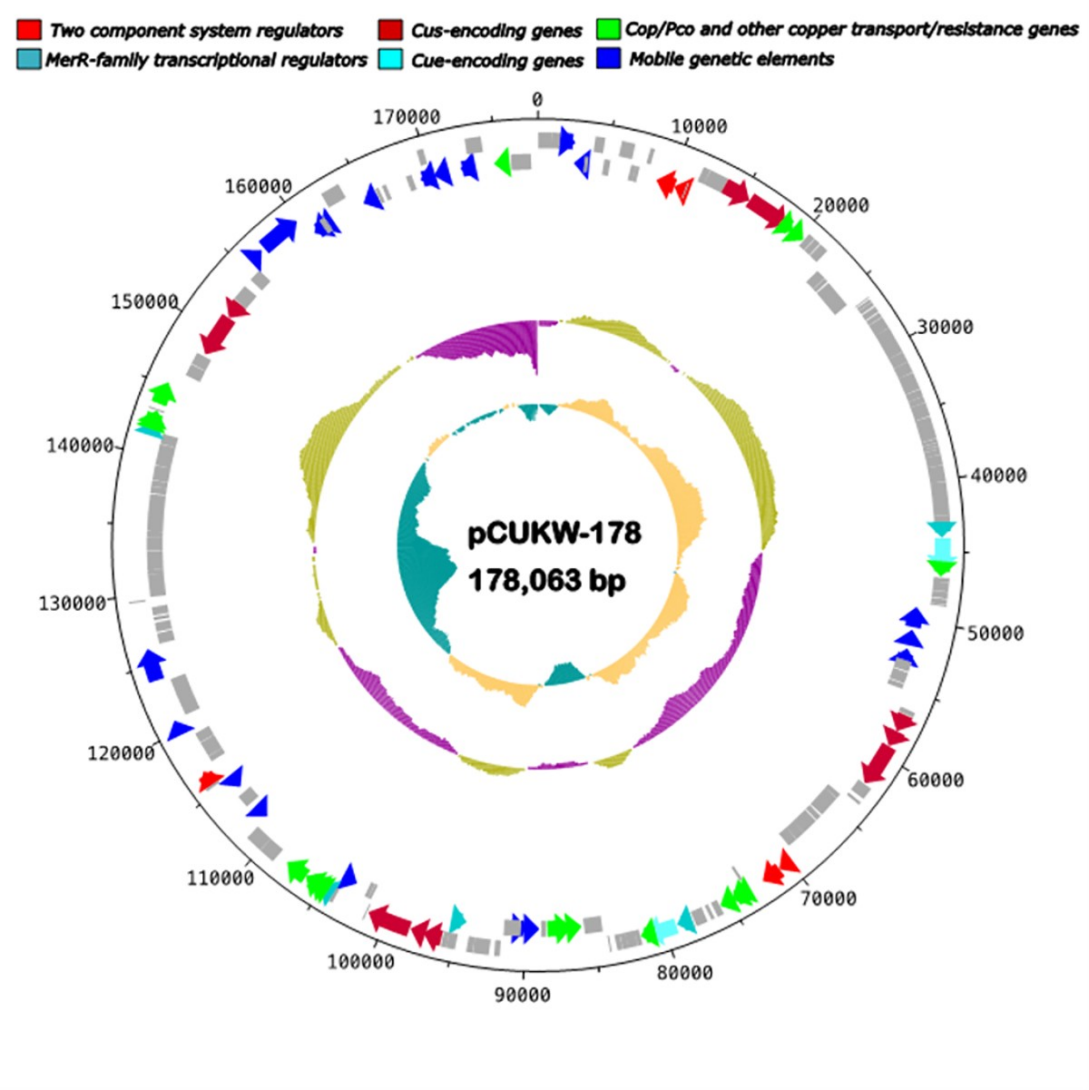
Genetic map of plasmid pCUKW-178. The rings (outer to inner) indicate CDSs on forward and reverse strand (ring 1 and ring 2), GC plot (ring 3), GC skew (ring 4). The CDSs are color coded as follows: mobile genetic element (dark blue), two component system regulators (red), MerR-family transcriptional regulator (dark aqua), Cus-encoding genes (dark red), Cue-encoding genes (aqua), Cop/Pco and other copper transport/resistance genes (bright green). The value of the GC plot is shown as: green for G+C content above average level and violet for G+C content below average level. The value of GC skew shows over abundance in yellow and under abundance in blue.

The genome content and organization of the plasmids was also examined (S9 Table). As reported previously, multiple genes associated with copper resistance were found to occur on plasmid pCUKW-178. The genes associated with the regulatory systems CueR and CopRS/PcoRS/CusRS were identified near the copper resistant genes in the plasmid. Genomic analysis indicated the presence of multiple mobile elements throughout the plasmid. However, there was no colocalization of mobile genetic elements and copper resistant clusters (Fig 9). The GC content plot displayed no dramatic changes near the copper resistant genes (Fig 9). Thus, there was no clear evidence showing a recent horizontal transfer of these copper-associated genes.

Further analysis was conducted with the *merR-copA* and *cusSR* genes. The genomic neighborhoods of both *cusSR* and *merR-copA* were analyzed using the Enzyme Function Initiative Genome Neighborhood Network server [34]. In both cases we observed evidence of moderate neighborhood reorganization, consistent with a vertical evolutionary process, in the genomic neighborhood of these operons (S8 and S9 Figs). This suggests that these operons have not been recently incorporated into the *Alteromonas* plasmids reported in this work.

GIs, along with other mobile genetic elements, contribute to the bacterial genome plasticity of a bacterial population. GIs typically bestow traits that enable enhanced adaptability within an environmental niche [64, 65]. They are widely distributed across pathogenic, non-pathogenic, and environmental microbes, where they are associated with pathogenicity, symbiosis, antibiotic resistance, xenobiotic degradation, and primary and secondary metabolism [64]. Colonization of copper-treated marine vessels requires the ability of the early colonizers to overcome the copper challenge, thus requiring adaptability to copper levels above those encountered in seawater. With regards to bacterial physiology, copper toxicity is increased when oxidized from the Cu(II) to Cu(I) state likely due to the ability of Cu(I) to diffuse through the cytoplasmic membrane [66, 67]. The potential for oxidation of the free cupric ion and the resulting Cu(I) challenge is enhanced in the colonization of copper coatings, as copper is oxidized under anaerobic conditions [66], such as would occur within the biofilm. The ability of *A*. *macleodii* CUKW *and* KCC02 to grow at copper levels lethal to other marine bacteria combined with the results of genomic and transcriptomic analyses indicate they are able to adapt and tolerate copper challenges via a flexible genome, as evidenced by the numerous copper homologs and the organization of key homologs such as *copA* on the plasmid and within a GI.

## Supporting information

S1 Fig Regardless of e-value, *Alteromonas* shows consistent enrichment of copper genes across systems, with representatives from all systems. (TIF)

S2 Fig A positive correlation exists across marine genera between decreasing e-value and number of copper-associated hits. (TIF)

S3 Fig *Alteromonas* CUKW and KCC02 show consistent enrichment across copper systems regardless of e-value. (TIF)

S4 Fig *Alteromonas* CUWK and KCC02 show consistent enrichment across systems regardless of e-value. (TIF)

S5 Fig Consistent enrichment of copper orthologs in *Alteromonas* CUKW and KCC02 regardless of e-value. (TIF)

S6 Fig Consistent enrichment in *Alteromonas* CUKW and KCC02 across copper systems regardless of e-value. (TIF)

S7 Fig Locations of genomic islands identified via IslandViewer on CUKW chromosome. (TIF)

S8 Fig Analysis of the genomic neighborhood surrounding the two *copA* variants across multiple bacterial species. (TIF)

S9 Fig Analysis of the genomic neighborhood surrounding *CusS* across multiple bacterial species. (TIF)

S1 Table All CUKW copper-associated genes identified based on an e-value cut-off of 10−30. (XLSX)

S2 Table All KCC02 copper-associated genes identified based on an e-value cut-off of 10−30. (XLSX)

S3 Table RefSeq accession numbers for marine species used in copper analysis. (XLSX)

S4 Table Hits to copper systems for all model species based on an e-value cut-off of 10−30. (XLSX)

S5 Table All copper-associated hits in CUKW across a range of e-values. (XLSX)

S6 Table All copper-associated hits in KCC02 across a range of e-values. (XLSX)

S7 Table Genes occurring on GI of pCUKW-178. The *copA* cluster is bolded.
(XLSX)

S8 Table List of genes located within each genomic island on the CUKW chromosome. (XLSX)

S9 Table List of genes illustrated on plasmid pCUKW-178. (XLSX)
