# Supplementary figures and images for: Enhanced copper-resistance gene repertoire in Alteromonas macleodii strains isolated from copper-treated marine coatings
Source: PLoS One. 2021 Sep 28;16(9):e0257800. doi: 10.1371/journal.pone.0257800 (PMC8478169; doi:10.1371/journal.pone.0257800)

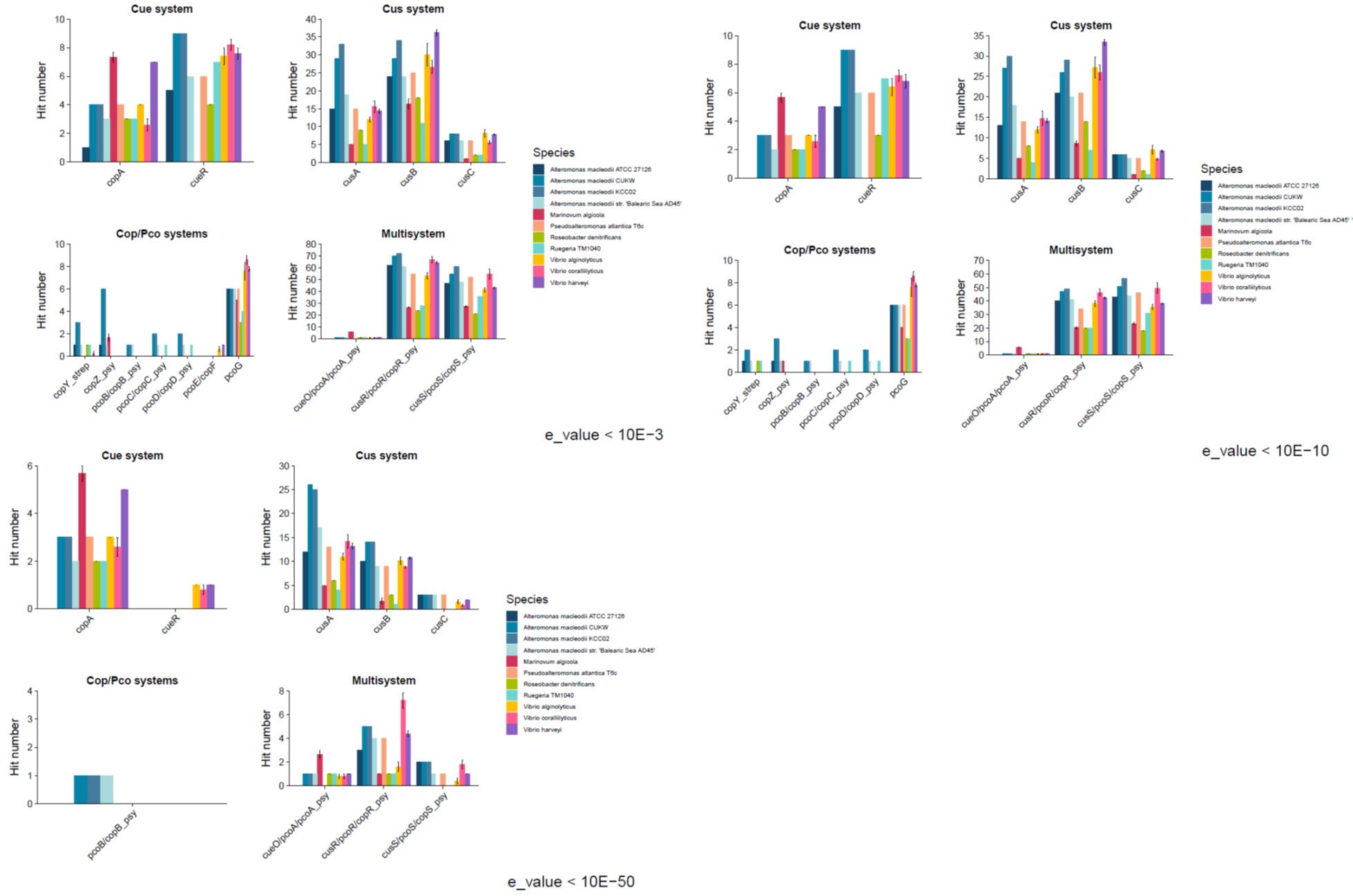

Supplement: S1 Fig — (TIF) [file pone.0257800.s001.tif]

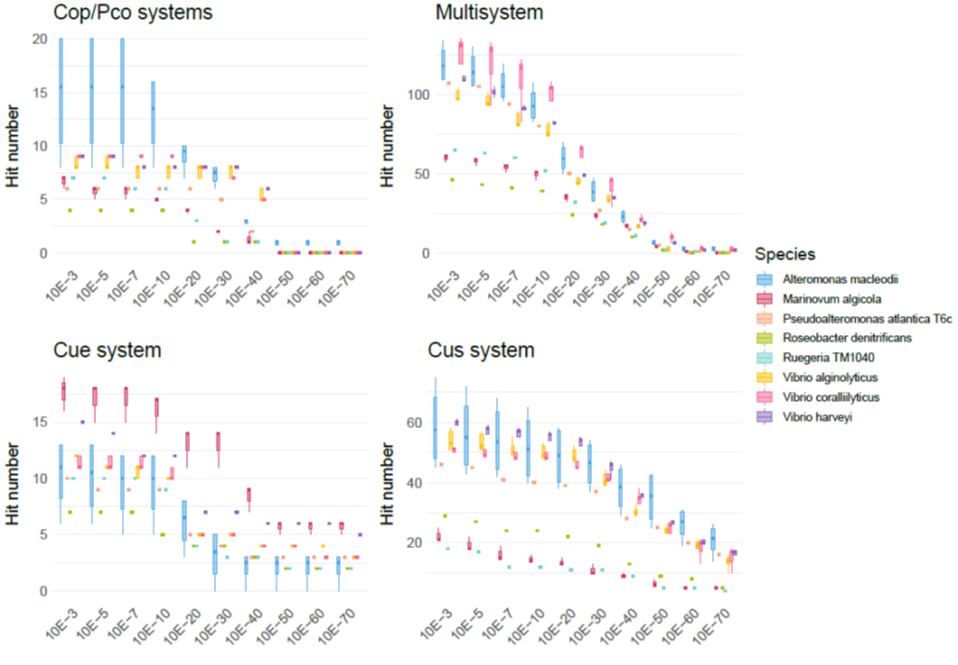

Supplement: S2 Fig — (TIF) [file pone.0257800.s002.tif]

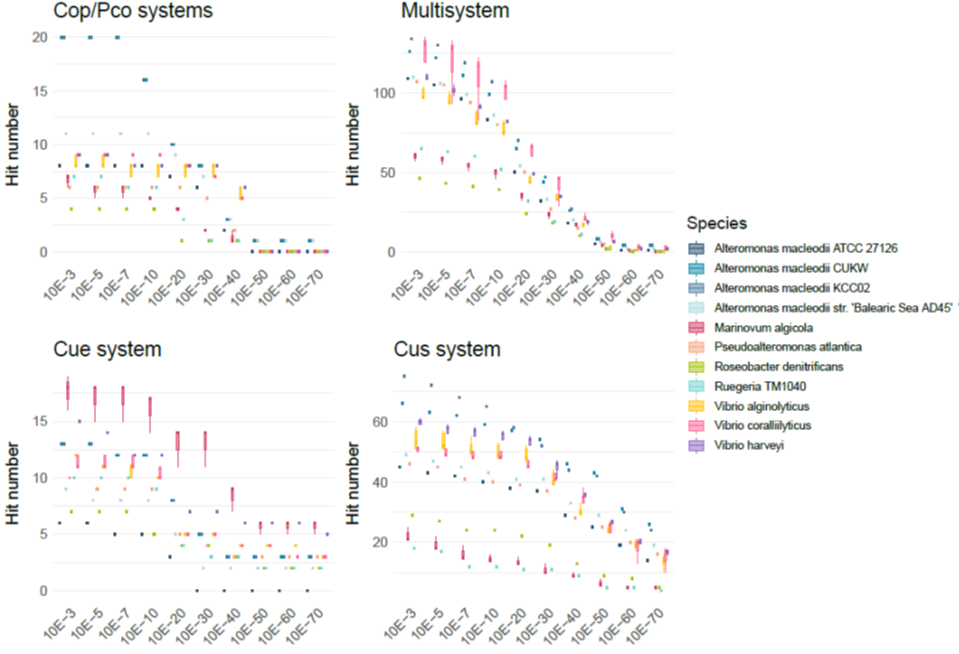

Supplement: S3 Fig — (TIF) [file pone.0257800.s003.tif]

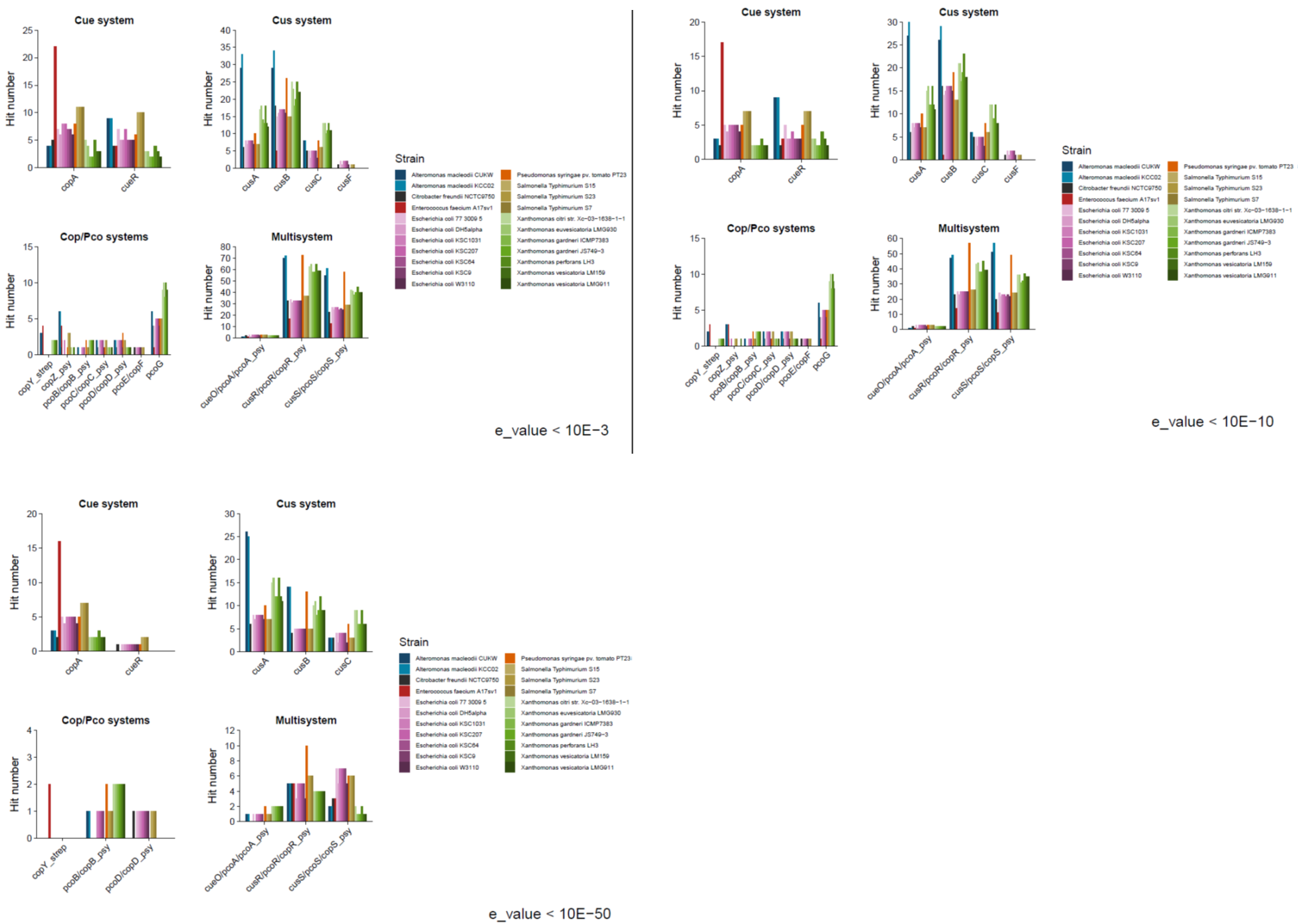

Supplement: S4 Fig — (TIF) [file pone.0257800.s004.tif]

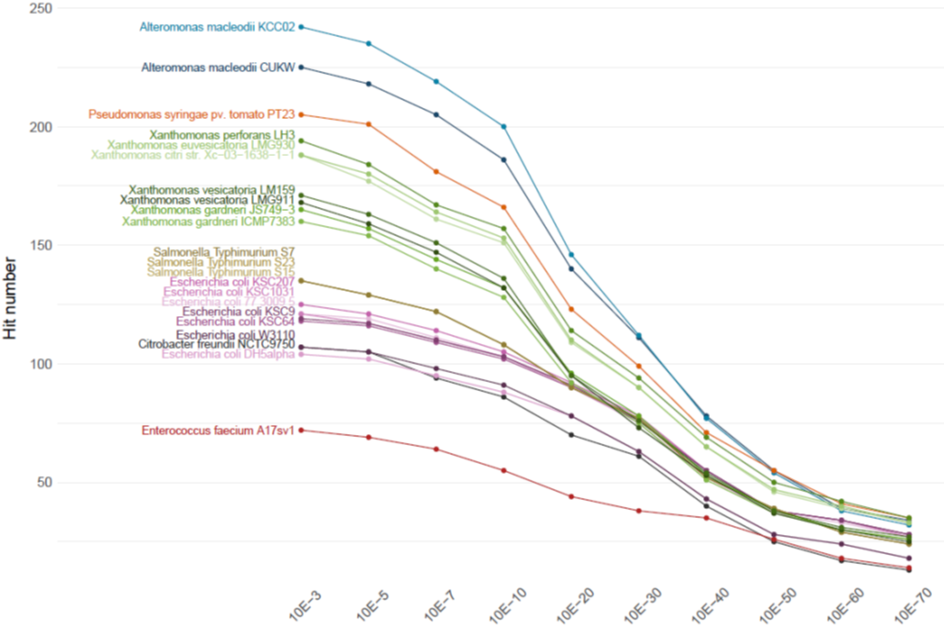

Supplement: S5 Fig — (TIF) [file pone.0257800.s005.tif]

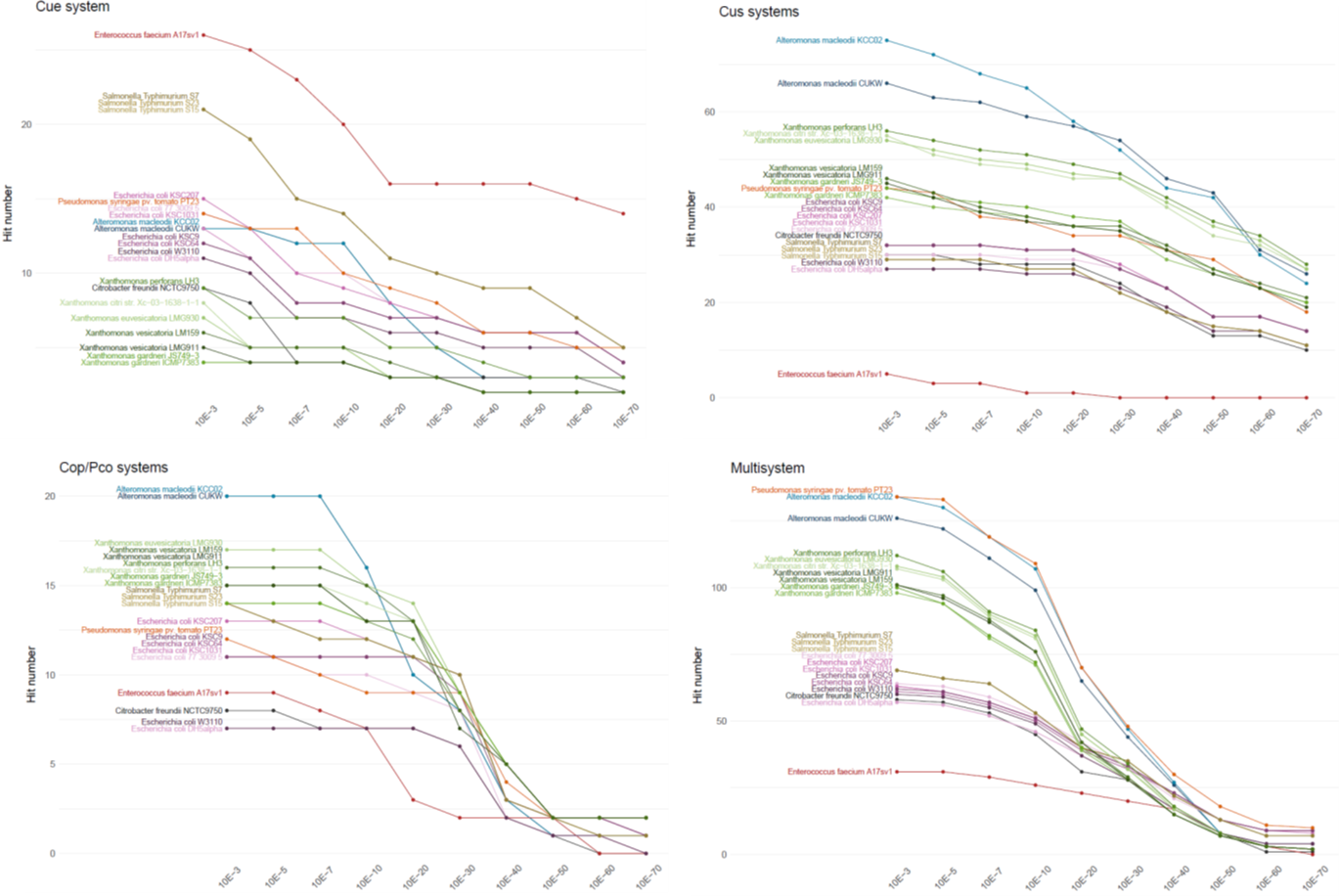

Supplement: S6 Fig — (TIF) [file pone.0257800.s006.tif]

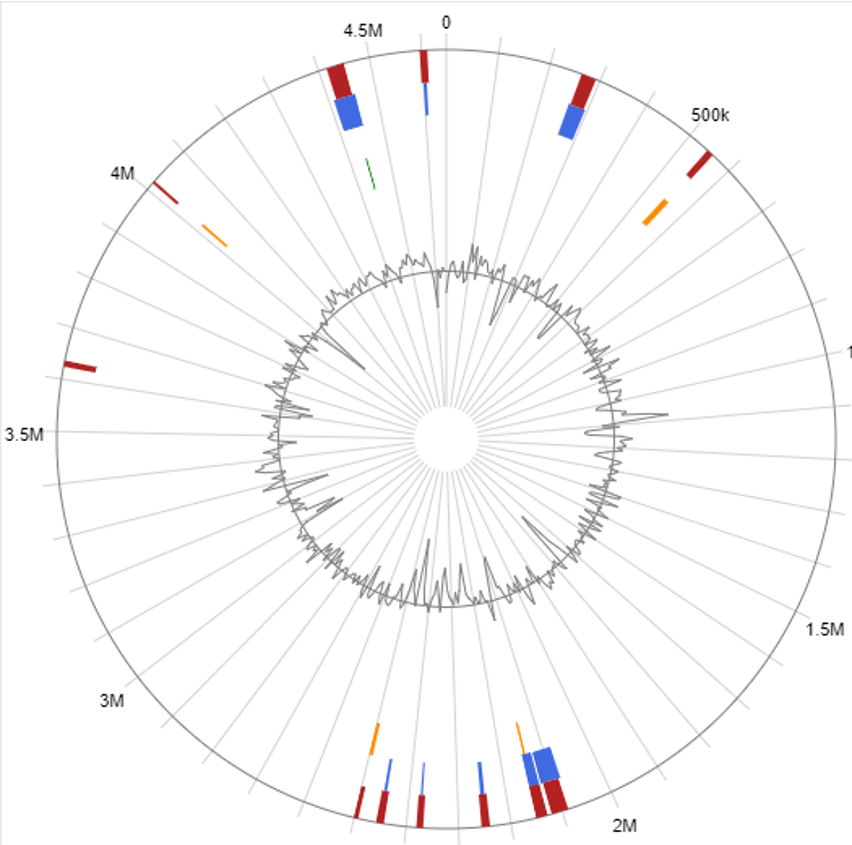

Supplement: S7 Fig — (TIF) [file pone.0257800.s007.tif]

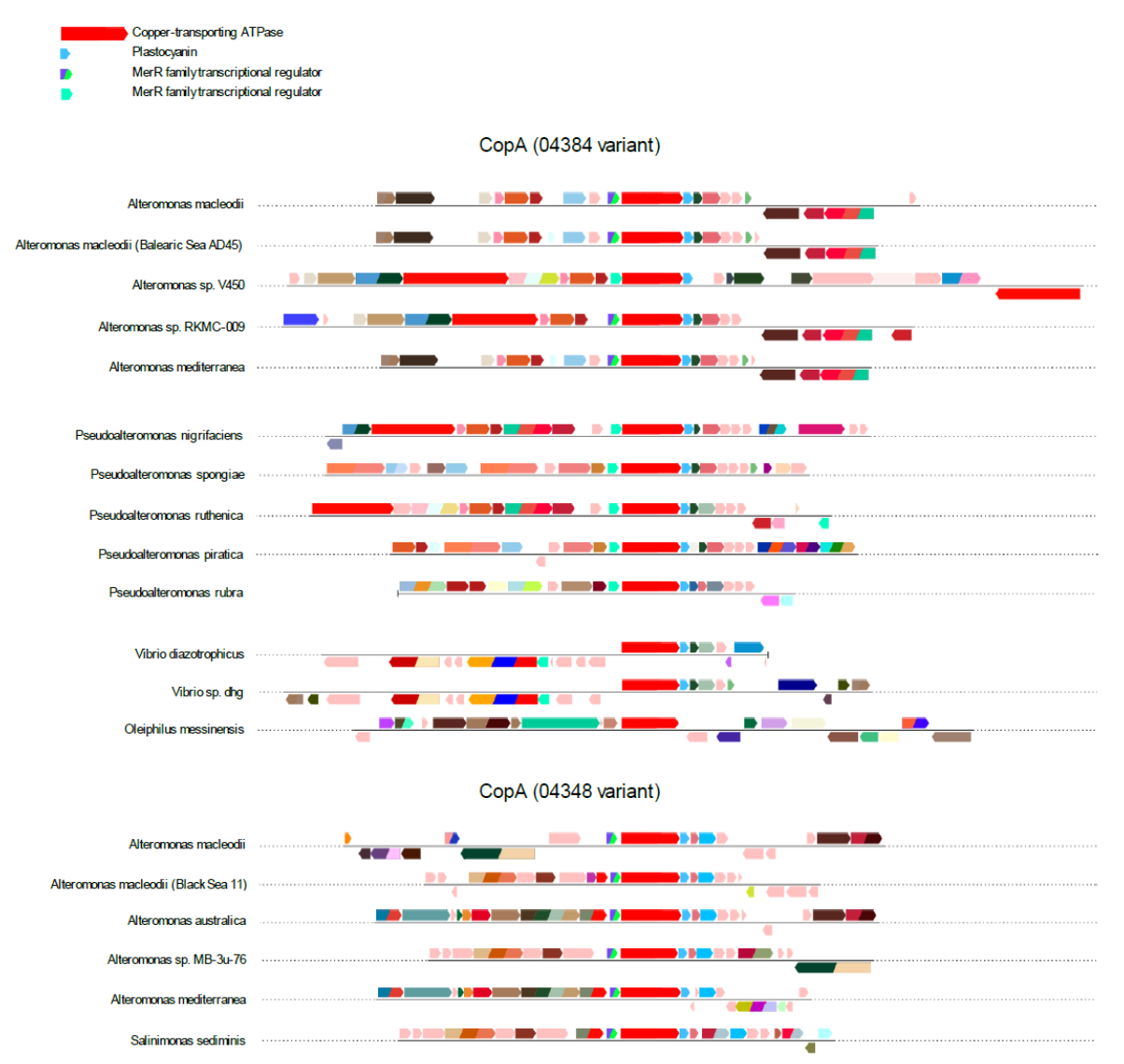

Supplement: S8 Fig — (TIF) [file pone.0257800.s008.tif]

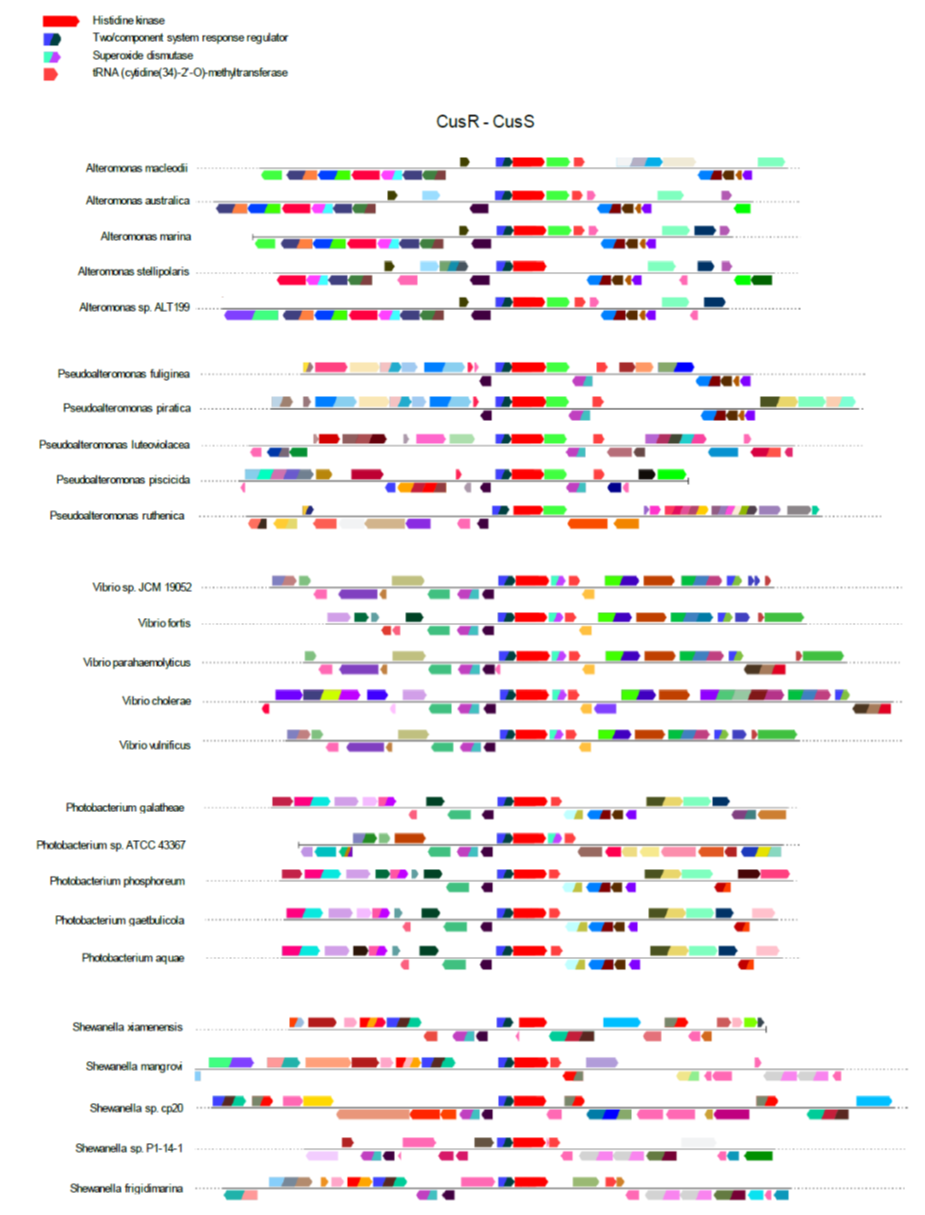

Supplement: S9 Fig — (TIF) [file pone.0257800.s009.tif]
